# High‐Performance Rechargeable Lithium‐Chlorine Batteries with ALD Conformal Starburst Porous Graphene Positive Electrodes

**DOI:** 10.1002/advs.202503113

**Published:** 2025-06-23

**Authors:** Zhuo Yang, Yanan Huang, Weicheng Zhou, Hong Fan, Zhihao Ding, Xu Yan, Yu Lu, Alexander S. Sigov, Wei Huang, Lijun Gao, Cheng Huang

**Affiliations:** ^1^ Soochow Institute for Energy and Materials InnovationS (SIEMIS) Key Laboratory of Advanced Carbon Materials and Wearable Energy Technologies of Jiangsu Province Key Laboratory of Core Technology of High Specific Energy Battery and Key Materials for Petroleum and Chemical Industry College of Energy Soochow University 688 Moye Road Suzhou 215006 P. R. China; ^2^ Physics and Energy Department Volta and DiPole Materials Labs International Joint MetaCenter for Advanced Photonics and Electronics School of Optical and Electronic Information Suzhou City University 1188 Wuzhong District Suzhou 215006 P. R. China; ^3^ Institute of Advanced Materials and Institute of Membrane Science and Technology Jiangsu National Synergistic Innovation Center for Advanced Materials State Key Laboratory of Flexible Electronics Suzhou Laboratory and Nanjing Tech University Nanjing 211816 P. R. China; ^4^ School of Flexible Electronics & State Key Laboratory of Optoelectronic Materials and Technologies Sun Yat‐sen University 66 Gongchang Road, Guangming District Shenzhen 518107 P. R. China; ^5^ Pacific Northwest National Laboratory Richland WA 99352 USA

**Keywords:** atomic layer deposition, conformal metasurface, dynamic respiratory interface, intelligent thermal management, rechargeable alkali metal‐chlorine battery, starburst porous graphene, ultrahigh‐capacity cathode

## Abstract

Rechargeable alkali metal‐chlorine batteries are emerging as a promising high‐energy‐density solution. However, they confront significant challenges, including the primary issue stemming from the weak binding affinity of cathode materials for Cl_2_, which leads to a sluggish and inadequate supply of Cl_2_ during the redox reactions, resulting in a shortened cycle life and low Coulombic efficiency (CE), particularly when operating at ultrahigh specific capacity outputs. Herein, an Al_2_O_3_‐skinned heterostructured starburst porous graphene with conformal metasurfaces (Al_2_O_3_@rGO) is reported, crafted from a hierarchical porous starburst graphene arranged in a unique layered structure by the PTFE microemulsion skin effect, leveraging subsequent fluidized bed atomic layer deposition (FBALD) of Al_2_O_3_ groups. Al_2_O_3_@rGO features superhydrophilicity, effective adsorption, fast kinetics from stable dynamic respiratory interface, high electrical and thermal conductivity anisotropy, intelligent thermal management and safe operation over a wide temperature range. Consequently, the Li‐Cl_2_@Al_2_O_3_@rGO battery achieves an ultrahigh discharge specific capacity of 5000 mAh g^−1^ at ≈100% CE, and even delivers stable cycling over 200 cycles with 2000 mAh g^−1^ at an average CE of 99.8% under low temperature environment of ‐40 °C. The scalable heterostructure approach offers a sustainable perspective of the development of functionalized metamaterials and metasurfaces for next‐generation safe and energy‐dense batteries and broader applications.

## Graphical abstract

An Al_2_O_3_‐skinned starburst porous graphene with conformal metasurfaces is crafted from a hierarchical porous graphene with high surface pore curvature by PTFE microemulsion skin effect, leveraging subsequent atomic layer deposition of Al_2_O_3_ groups. The scalable heterostructured cathode features superhydrophilicity, effective adsorption, fast kinetics from stable dynamic respiratory interface, high electrical/thermal conductivity anisotropy, achieving ultrahigh specific capacities and intelligent thermal management.

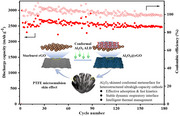

## 1 Introduction

The development of sustainable batteries with high energy density has become crucial to accommodate the escalating demands.^[^ ^1^, ^2^, ^3^, ^4^, ^5^, ^6^, ^7^ ^]^ Primary lithium‐thionyl chloride (Li‐SOCl_2_) batteries have garnered significant interest due to their high operating voltage, high energy density, and a broad operating temperature range, leading to their extensive application across aviation, aerospace, military, electronics, and other sectors. In 2021, Dai et al. pioneered the development of rechargeable Li‐Cl_2_ batteries, building upon the foundation of Li‐SOCl_2_ batteries and significantly broadening their potential applications.^[^ ^8^, ^9^, ^10^, ^11^, ^12^, ^13^, ^14^, ^15^, ^16^, ^17^, ^18^, ^19^ ^]^ A rechargeable Li‐Cl_2_ battery typically consists of a Li metal anode, a non‐aqueous electrolyte based on SOCl_2_, and a carbon cathode material. It offers a discharge voltage of up to 3.5 V and a high specific capacity reaching 1200 mAh g^−1^, positioning them as one of the most advanced battery energy storage systems currently available. Nevertheless, several hurdles must be cleared to facilitate the practical deployment of high‐energy‐density Li‐Cl_2_ batteries.^[^ ^18^, ^20^, ^21^ ^]^ The primary challenge of rechargeable alkali metal‐chlorine batteries is the inadequate supply of Cl_2_ during the reaction phase, stemming from the weak affinity between the cathode material and Cl_2_ molecules. Additionally, the shuttling effect of unbonded Cl_2_ can cause battery decay, particularly at a high output capacity. Consequently, materials featuring a porous structure are pivotal in addressing these issues, and the pore architecture can be enhanced by incorporating polar groups capable of engaging with Cl atoms.^[^ ^18^ ^]^

Until now, cathode materials for Li‐Cl_2_ batteries encompass a variety of options, including carbon materials,^[^ ^11^, ^15^, ^16^, ^17^ ^]^ metal‐organic frameworks (MOFs),^[^ ^20^ ^]^ and porous organic cages (POCs).^[^ ^21^ ^]^ Dai et al. have explored the utilization of amorphous carbon nanospheres (aCNS) and defective graphite as cathode materials for Li‐Cl_2_ batteries.^[^ ^15^, ^17^ ^]^ However, these materials have demonstrated low cycling specific capacity, weak physical adsorption of Cl atoms on the carbon surface, and a lack of effective Cl_2_ trapping capability. Chen et al. have reported on the use of metal‐organic frameworks (MOFs) as cathodes for Li‐Cl_2_ batteries,^[^ ^20^ ^]^ but the existence of the metal in MOFs increased the weight and instability risk of the Li‐Cl_2_ batteries. Furthermore, our group has also investigated the application of functionalized porous organic nanocages (POCs) as efficient porous microreactors for capturing Cl_2_ gas.^[^ ^21^ ^]^ However, these organic electrode materials are plagued by poor electrical conductivity, high solubility in the electrolyte, and low density.^[^ ^22^ ^]^ Graphene boasts a suite of superior attributes, including high energy density, exceptional mechanical properties, outstanding electrical conductivity, excellent chemical stability, and remarkable thermal conductivity.^[^ ^23^, ^24^ ^]^ Owing to these inherent characteristics, graphene has been successfully integrated into a multitude of applications, ranging from supercapacitors and lithium‐ion batteries to electronic components.^[^ ^25^, ^26^, ^27^ ^]^ For instance, Zhang et al. detailed the use of hierarchical porous graphene as an electrode in lithium‐air batteries,^[^ ^28^ ^]^ which significantly enhanced the specific capacity of batteries. Motivated by these findings, we set our sights on investigating functionally graded porous graphene as electrode scaffolds for the development of ultrahigh‐capacity cathodes of rechargeable alkali metal‐chlorine batteries and their underlying mechanism while it has not yet been revealed up until now. In addition, by atomic layer controlled growth, an atomic layer deposition (ALD) process provides a sophisticated surface chemistry technique and enhanced 3D additive manufacturing where precursor gases are introduced into the reactor in a sequential manner, with inert gases employed to cleanse the separation pulse.^[^ ^29^, ^30^, ^31^, ^32^ ^]^ By supplying different types of precursors in an alternating fashion, the deposition and growth of materials can be meticulously controlled at the nanoscale, particularly with atomic‐level thicknesses for atomic‐level manufacturing.^[^ ^33^, ^34^, ^35^ ^]^ Especially in the FBALD process of an atomic layer manufacturing, the feedstock in the fluidized state can be fully contacted in the precursor, thus ensuring the uniformity of the deposited material at the atomic or molecular level to solve the conformal issue of 3D curved surfaces for scalable ALD of layered heterostructured electrodes with conformal metasurfaces.

To address the challenge of developing ultrahigh‐capacity cathode materials for high‐performance rechargeable alkali metal‐chlorine batteries, we propose employing starburst graphene with a hierarchical porous structure and inclined homeotropic or vertical nanosheet alignment, enhanced by the atomic layer deposition of Al_2_O_3_ on its surface, as a means to enrich Cl_2_ gas and thereby achieve the high CE, extended cycle life, and elevated discharge specific capacity essential for Li‐Cl_2_ batteries. Herein, we report ALD Al_2_O_3_‐skinned layered heterostructured electrodes with conformal metasurfaces for ultrahigh‐capacity cathodes. A starburst reduced graphene oxide (rGO) was fabricated by the PTFE colloidal microemulsion skin effect, and subsequently Al_2_O_3_ atomic layer epitaxy was applied onto the starburst rGO surface (Al_2_O_3_@rGO) through FBALD, leveraging the precise control over film thickness and uniformity that FBALD offers. As a proof, the density‐functional theory (DFT) calculation was conducted to confirm the robust interaction between Al_2_O_3_ and Cl_2_ molecules, thereby validating the material ability to effectively enrich Cl_2_ gas. Moreover, the Al_2_O_3_ coating applied via FBALD not only enhances electron and ion conductivity but also stabilizes the structure of rGO material during battery cycling wearing a dense curved conformal thin film of Al_2_O_3_ armor to shield itself from further Cl_2_ corrosion. It further optimizes the material surface characteristics, thereby increasing the number of electrocatalytic active sites and boosting the reaction rate. The Al_2_O_3_@rGO demonstrated the excellent hydrophilicity, high electrical conductivity, high thermal conductivity anisotropy, intelligent thermal management and safe operation over a wide temperature range. The scalable heterostructure approach of this hierarchical starburst porous functionalized cathode material with Al_2_O_3_‐skinned conformal metasurfaces achieved high‐performance Li‐Cl_2_ battery systems with the features of effective adsorption and fast kinetics, stable dynamic respiratory interface, and intelligent thermal management. Upon employing the ALD conformal starburst porous graphene as the cathode material in Li‐Cl_2_ batteries, these batteries delivered an exceptional initial discharge specific capacity exceeding 12 000 mAh g^−1^ and maintained stable operation for approximately 180 cycles at a high discharge specific capacity of 2500 mAh g^−1^. Furthermore, the Li‐Cl_2_ batteries incorporating this material exhibited outstanding performance even at extremely low temperatures, down to ‐40 °C.

## 2 Results and Discussion

**Figure 1:**
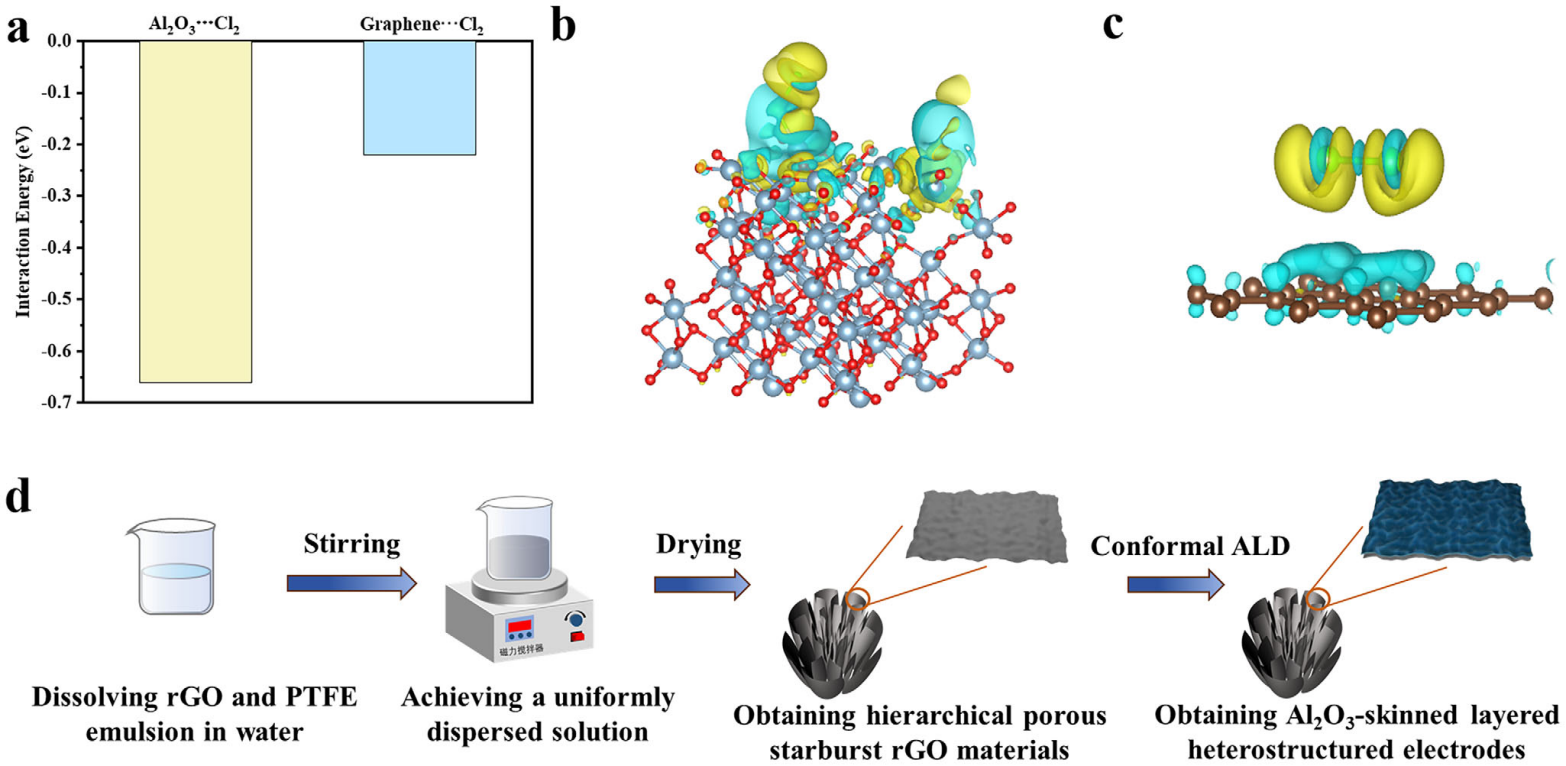
Calculation of adsorption energies and charge density difference of the Cl_2_ adsorption sites on pristine Al_2_O_3_ and graphene. a) Adsorption energies of Al_2_O_3_ and graphene to Cl_2_. Charge density difference plots of b) Al_2_O_3_ and c) graphene repetitive units with respect to Cl_2_. The yellow zone represents the electron‐absorbing region, and the blue zone represents the electron‐loss region. d) Schematic diagram of the Al_2_O_3_@rGO heterostructure preparation via the microemulsion skin effect and subsequent conformal FBALD.

In order to clearly illustrate the chemisorption state of Al_2_O_3_ for Cl_2_, the first‐principles calculations based on density‐functional theory (DFT) were used to determine the magnitude of the adsorption energy of Cl atoms on the Al_2_O_3_ moiety, and graphene with a typical sp^2^ structure was chosen as a comparative illustration. As shown in **Figure** **1**a, the adsorption energy of Al_2_O_3_ for Cl_2_ is −0.66 eV, which was much lower than that of graphene at −0.22 eV, indicating a stronger affinity for Cl_2_ by Al_2_O_3_. Figure S1 (Supporting Information) illustrates the optimized structural units for Cl_2_ adsorption on Al_2_O_3_@rGO and graphene. Furthermore, the Cl_2_ adsorption sites on Al_2_O_3_ and graphene were investigated using charge density difference plots. Figure 1b,c presents the charge density difference plots for the adsorption of Cl_2_ by Al_2_O_3_ and graphene, respectively. In contrast to the adsorption by graphene, the adsorption by Al_2_O_3_@rGO results in a more extensive electronic departure domain around the deposited Al_2_O_3_, suggesting that the adsorption sites are predominantly near the Al atoms and that the adsorption intensity of Al_2_O_3_ is considerably stronger. It is mentioned that there are some structural and property differences between rGO and perfect graphene considering the concentration of the lattice defects and functional groups such as C/O, depending on the materials preparation and processing control. However, the simulation simplification does not significantly influence the comparison between polar Al_2_O_3_ and rGO with adsorbed Cl_2_ based on the experimental results and assisted by the early calculations.^[^ ^28^ ^]^ The flowchart in Figure 1d outlines the synthesis process of Al_2_O_3_@rGO via the microemulsion skin effect and subsequent conformal FBALD. rGO was initially dispersed within an aqueous PTFE microemulsion. After thorough mixing and homogenization, the mixture was then cast and dried to form a hierarchical porous starburst rGO material which is inclined towards vertical graphene nanosheets employing the colloidal microemulsion technique and the skin effect, followed by deposition of a thin film of Al_2_O_3_ on its surface using FBALD to obtain the conformal Al_2_O_3_@rGO layered heterostructured material, an Al_2_O_3_‐skinned starburst porous electrode with conformal metasurfaces.

**Figure 2:**
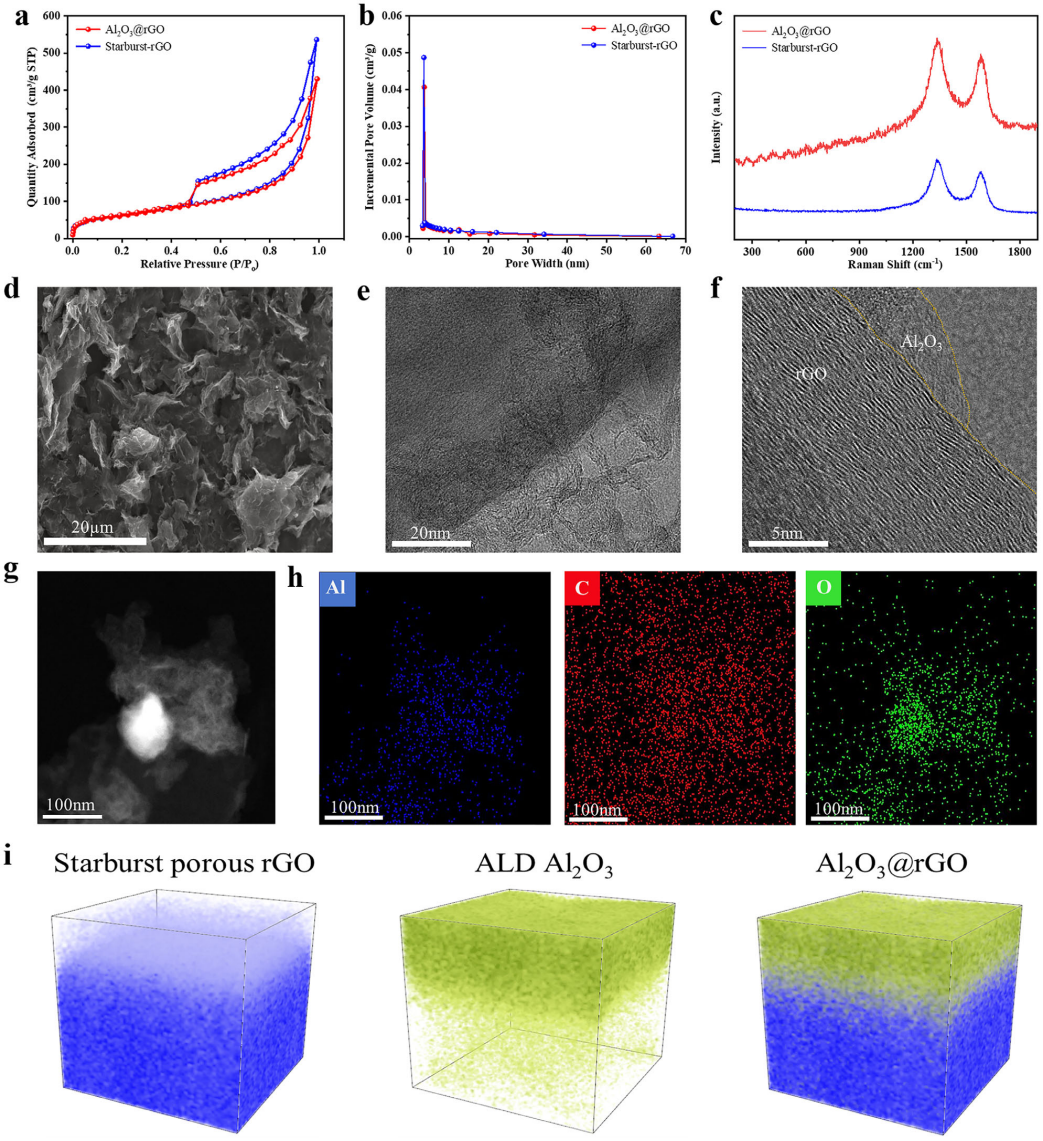
a) N_2_ adsorption/desorption isotherms of Al_2_O_3_@rGO and starburst porous rGO at 77 K and b) BET analysis and the fitted pore size distribution of Al_2_O_3_@rGO and starburst porous rGO from the N_2_ adsorption isotherm. c) Raman spectra of Al_2_O_3_@rGO and starburst porous rGO. d) SEM image of prepared starburst porous rGO. e,f) High‐resolution TEM images of Al_2_O_3_@rGO heterostructure. g) STEM image of Al_2_O_3_@rGO heterostructure and h) STEM‐EDX elemental mapping images of Al_2_O_3_@rGO heterostructure. i) 3D distributions (analysis area: 50 × 50 µm^2^) of rGO, Al_2_O_3_ and Al_2_O_3_@rGO secondary ion fragments reconstructured from TOF‐SIMS depth profiling of an Al_2_O_3_@rGO electrode.

N_2_ adsorption‐desorption experiments were conducted at 77 K to analyze the pore structures of both starburst porous rGO and Al_2_O_3_@rGO. As illustrated in **Figure** **2**a, the N_2_ adsorption‐desorption isotherm exhibited type‐IV characteristics with distinct hysteresis loops in the relative pressure range (*P*/*P*₀) of 0.5–1.0, indicating the presence of abundant mesopores in both materials. The pore size distributions reveal that the majority of pores are concentrated in the 3–10 nm range as shown in Figure 2b. The high density of small pores can be attributed to their hollow structures and rich surface defects. Additionally, the Brunauer‐Emmett‐Teller (BET) specific surface area of starburst porous rGO is 225 m^2^ g^−1^, while that of Al_2_O_3_@rGO is slightly reduced to 219 m^2^ g^−1^, which may result from the influence of the Al_2_O_3_ coating deposited via ALD technology. Raman spectroscopy was performed on the synthesized starburst porous rGO and Al_2_O_3_@rGO as depicted in Figure 2c. The peaks at ≈1350 cm⁻¹ and ≈1600 cm⁻¹ correspond to the D‐band (disordered carbon) and G‐band (graphitic carbon), respectively. The intensity ratio of the D‐band to G‐band (*I*_D_/*I*_G_) reaches 1.28, confirming the highly disordered nature of both materials. For Al_2_O_3_@rGO, the broad weak peaks observed at ≈420 cm^−1^, ≈500 cm^−1^, and ≈600 cm^−1^ likely originate from the amorphous Al_2_O_3_ deposited via atomic layer deposition. X‐ray diffraction (XRD) further confirmed the highly disordered state of the starburst rGO, with a broad peak centered at ≈26° (Figure S2, Supporting Information), which can be attributed to carbon. Figure S3 (Supporting Information) shows the optical images of starburst rGO and Al_2_O_3_@rGO under an upright fluorescence microscope. The synthesized starburst porous rGO was morphologically characterized using scanning electron microscopy (SEM). As shown in Figure 2d, the material of starburst porous rGO which tends towards vertical graphene by microemulsion skin effect features an extensive network of interconnected channel structures, adjacent to numerous smaller nanoscale pores that link to the larger channels. This distinctive architecture is an optimal design for the electrode in Li‐Cl_2_ batteries, facilitating the accommodation of LiCl/Cl_2_ during the redox process. The composite electrode Al_2_O_3_@rGO, after the deposition of Al_2_O_3_, was further examined using high‐resolution scanning transmission electron microscopy (HRSTEM) and energy dispersive X‐ray spectroscopy (EDX), as presented in Figure 2. The TEM images of Al_2_O_3_@rGO heterostructure (Figure 2e,f) reveal an amorphous layer of Al_2_O_3_ on the rGO surface (Figure 2f). The elemental analysis (Figure 2g,h) confirms the uniform deposition of Al_2_O_3_, as detailed in the Figure 2 and Figure S9 (Supporting Information), showcasing a complete fluidized bed atomic layer deposition (FBALD) coating on rGO. Furthermore, 3D chemical mapping of the prepared Al_2_O_3_@rGO composite electrode was reconstructured and performed using time of flight‐secondary ion mass spectrometry (TOF‐SIMS) depth profiling as shown in Figure 2i. In the composite electrode, high Al_2_O_3_ counts were detected and uniformly distributed across the starburst porous rGO substrate, confirming the successful and homogeneous deposition of the amorphous Al_2_O_3_ coating on the starburst porous rGO cathode via ALD (Figure S4, Supporting Information). In contrast to crystalline Al_2_O_3_, the amorphous Al_2_O_3_ layer possesses elasticity and buffering capabilities that help prevent structural alterations to the rGO during cell cycling.^[^ ^32^ ^]^ The ample reaction sites furnished by the deposited Al_2_O_3_ groups, coupled with the substantial specific surface area and porous architecture of rGO, render it an exemplary cathode material for the enrichment of Cl_2_ molecules. It is mentioned that the graphene surface pretreatment and the substrate‐interface interactions between deposited Al_2_O_3_ and underlying graphene, especially at 3D curved surface of the starburst porous graphene, influence the Al_2_O_3_ film growth mechanisms. Without O_3_ pretreatment, a porous insulator contact might occur at the interface by 3D island growth (Volmer‐Weber) model. With O_3_ pretreatment, a conformal buried interface dielectric passivation layer significantly occurs by 2D layer‐by‐layer growth (Frank‐van der Merwe) model and even by hybrid layer‐island growth (Stranski‐Krastanov) model. Therefore, completely clad in armor, atomic layer deposition of Al_2_O_3_ results in a conformal buried interface passivation layer within Al_2_O_3_@rGO heterostructure. The Al_2_O_3_‐skinned starburst graphene wearing a dense curved conformal thin film of Al_2_O_3_ armor, not only bolsters the structural stability of starburst porous rGO as electrode scaffolds during battery cycling, but also shields graphene from further corrosion with ALD Al_2_O_3_ layer as a sacrificial agent for Cl_2_ corrosion prevention, especially for the enrichment of Cl_2_ molecules within superhigh‐capacity cathode.

As comparisons, three distinct meta‐structured electrode samples (Pristine‐rGO, Starburst‐rGO and Al_2_O_3_@rGO) with anisotropy and degrees of freedom^[^ ^36^, ^37^ ^]^ as well as conformal metasurface were fabricated for further performance evaluation and the schematic representations of these materials structures are depicted in Figure S5 (Supporting Information). Pristine‐rGO is a traditional rGO textured electrode with inclined homogeneous or horizontal alignment (Figure S5a, Supporting Information). Starburst‐rGO is a starburst rGO hierarchical porous electrode with inclined homeotropic or vertical alignment by the PTFE colloidal microemulsion skin effect (Figure S5b, Supporting Information). Al_2_O_3_@rGO is an ALD Al_2_O_3_‐skinned conformal metasurface heterostructured electrode by the PTFE microemulsion skin effect and subsequent atomic layer epitaxy (Figure S5c, Supporting Information). Specifically, there are pristine‐rGO having a typical lamellar stacked structure, starburst‐rGO treated by the emulsion method exhibiting a hierarchically disordered starburst porous arrangement^[^ ^38^ ^]^ which facilitates ionic transport, and a thin Al_2_O_3_ film deposited on the surface of the starburst porous rGO using FBALD. The contact angle tests and hydrophilicity analysis were conducted for these three samples to simulate and evaluate electrolyte wettability or electrode infiltration. As shown in Figure S6 (Supporting Information), there are pristine‐rGO with hydrophobicity, starburst rGO with enhanced hydrophobicity, and Al_2_O_3_@rGO with superhydrophilicity. Al_2_O_3_@rGO conformal metasurface demonstrated the highest hydrophilicity among the samples, likely due to the co‐growth of Al_2_O_3_ on the rGO surface through the FBALD process. Furthermore, the thermal conductivity and heat dissipation of these three samples were tested to simulate and evaluate the prevention of battery thermal runaway at elevated temperatures and the battery low‐temperature insulation behaviors at different low temperatures. As illustrated in Figure S7 (Supporting Information), when placed on a heating table set to various elevated temperatures, the starburst rGO and Al_2_O_3_@rGO with surface‐deposited Al_2_O_3_ displayed higher temperatures, and they had a smaller temperature difference with the heating table, indicating better thermal conductivity. The starburst rGO and Al_2_O_3_@rGO are superior to Pristine‐rGO due to anisotropic thermal conductivities at the horizontal and vertical level of starburst rGO and Al_2_O_3_. This can be attributed to the 3D layered structure of the starburst rGO, which enhances heat conduction, and the inherent good thermal conductivity of Al_2_O_3_. The enhanced thermal conductivity effectively channels the heat generated within the battery, preventing thermal runaway at elevated temperatures and thereby enhancing the battery safety and lifespan. As shown in Figure S8 (Supporting Information), when tested under low temperature environment, at ‐10 °C and ‐20 °C, the temperature of starburst rGO is the lowest, and the temperatures of pristine‐rGO and starburst rGO coated with Al_2_O_3_ are higher. Low‐temperature insulation behaviors of Al_2_O_3_@rGO and pristine‐rGO are superior to starburst rGO due to anisotropic thermal conductivities at the horizontal and vertical level of pristine‐rGO and Al_2_O_3,_ which indicates that the temperatures can be maintained better under low temperature environment after depositing Al_2_O_3_ on the basis of starburst rGO, which is conducive to the cycling of battery at low temperature. The Al_2_O_3_@rGO composites, engineered through atomic layer deposition, possess a 3D open interlayer structure, achieving excellent hydrophilicity, high electrical conductivity, high thermal conductivity anisotropy, intelligent thermal management and safe operation over a wide temperature range, by simultaneously optimizing the anisotropic transport properties of the electrical (electron and ion) and thermal conductivities. These characteristics suggest that they hold significant potential for applications in energy storage batteries.

**Figure 3:**
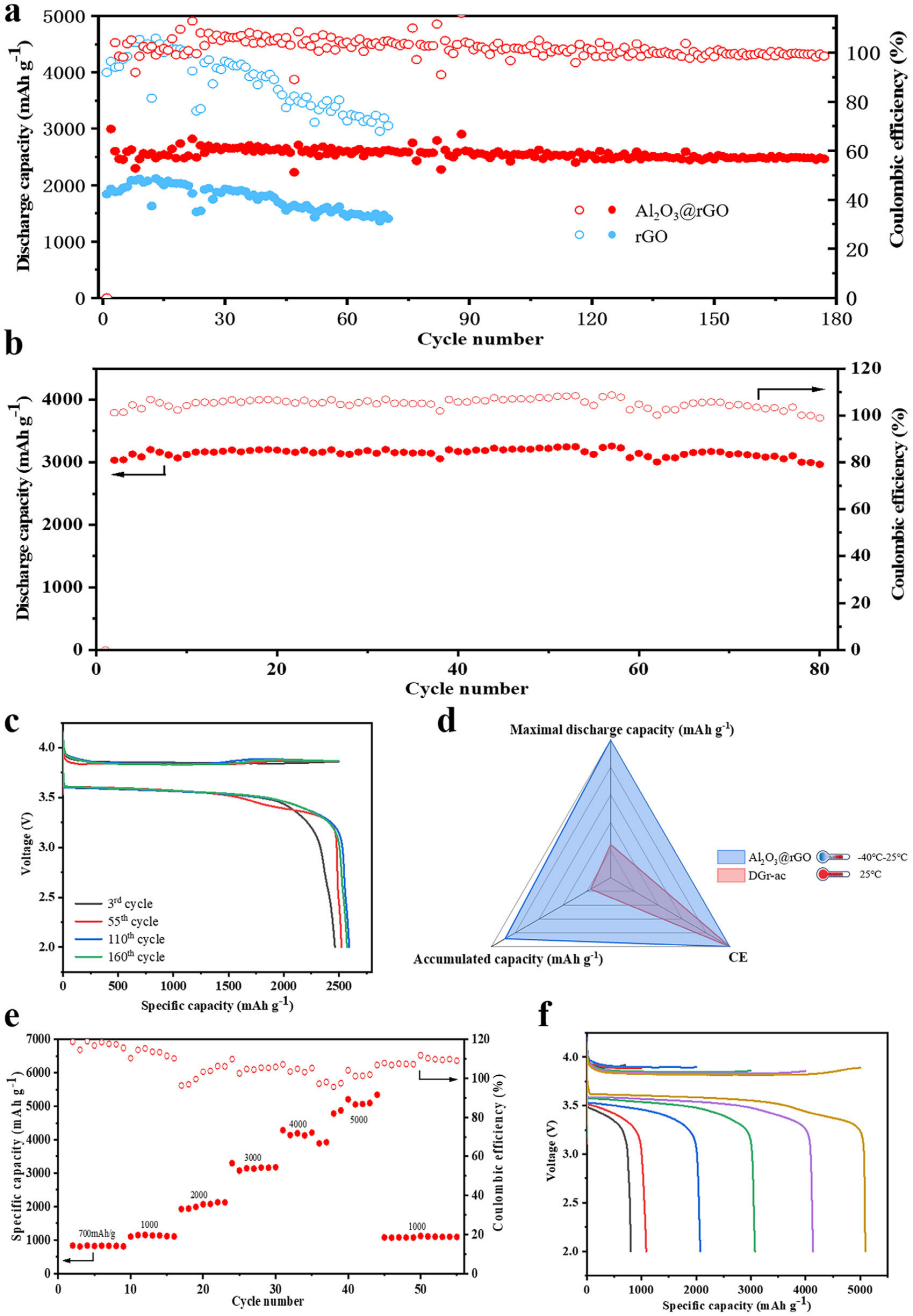
Electrochemical performances of the Li−Cl_2_@Al_2_O_3_@rGO cell under high cycling specific capacities. a) Cycling performance of the Li−Cl_2_ cell using Al_2_O_3_@rGO and starburst rGO cathodes at charge capacities of a) 2500 mAh g^−1^ and b) 3000 mAh g^−1^. c) Voltage profiles of the Li−Cl_2_@Al_2_O_3_@rGO cell under a charge capacity of 2500 mAh g^−1^ at different cycles. d) Comparison of this work with the previously reported DGr‐ac in terms of CE, accumulated capacity, and maximal discharge capacity. e) Cycling performance and (f) voltage profiles of the Li−Cl_2_@Al_2_O_3_@rGO cell under charge capacities from 700 to 5000 mAh g^−1^.

To estimate the capacity of Al_2_O_3_@rGO to enrich Cl_2_ under electrochemical conditions, Li‐Cl_2_@Al_2_O_3_@rGO batteries were assembled and subjected to the cycling tests at elevated specific capacities. This assessment was crucial for understanding the impact of Al_2_O_3_@rGO on the conversion reactions involving LiCl and Cl_2_ gas. Initially, the Al_2_O_3_@rGO electrode was characterized using scanning electron microscopy (SEM) and the corresponding energy dispersive X‐ray spectroscopy (EDX). As shown in Figure S9 (Supporting Information), the Al_2_O_3_@rGO was uniformly distributed across the conductive carbon, as the C, Al elements were evenly distributed. It is worth mentioning that specific capacities of the batteries were calculated based on the mass of Al_2_O_3_@rGO. As shown in Figure S10 (Supporting Information), the initial discharge capacity of the fabricated Li‐Cl_2_@Al_2_O_3_@rGO battery exceeds 12 000 mAh g^−1^, significantly outperforming the 3309 mAh g^−1^ reported in the literature. In addition, the electrochemical impedance EIS spectra analysis of the two electrodes, starburst rGO and Al_2_O_3_@rGO, for the initial conditions (Figure S11, Supporting Information) indicates that Al_2_O_3_@rGO still exhibits the high electrical conductivity nature of rGO and quantum (electron) tunneling behavior of the atomic layer even Al_2_O_3_ ALD on the rGO surface. This exceptionally high initial discharge capacity suggests that the specific surface area, porosity, and active sites of Al_2_O_3_@rGO have been markedly enhanced, allowing for greater accommodation of LiCl. As illustrated in **Figure** **3**a, the Li‐Cl_2_@Al_2_O_3_@rGO battery was charged at an ultra‐high capacity of 2500 mAh g^−1^ with a current density of 800 mA g^−1^. After ≈180 cycles, the Li‐Cl_2_@Al_2_O_3_@rGO battery maintained a substantial discharge capacity of 2465 mAh g^−1^ with a CE of 98.6%. Even under the conditions that the charging capacities of the battery were elevated to 2800 and 3000 mAh g^−1^, respectively, the cycle life of the Li‐Cl_2_@Al_2_O_3_@rGO battery extended to approximately 100 and 80 cycles, respectively, with Coulombic efficiencies of 93% and 99% (Figure S12, Supporting Information and Figure 3b). These results underscore the exceptional LiCl storage capacity and enduring cycle life of our engineered Li‐Cl_2_@Al_2_O_3_@rGO cell, demonstrating that Al_2_O_3_@rGO possesses superior adsorptive and storage capabilities for Cl_2_. At a cycling capacity of 2500 mAh g^−1^, the discharge plateau surpassed 3.5 V, and a stable, flat discharge voltage plateau was sustained over 160 cycles (Figure 3c). This further suggests that Al_2_O_3_@rGO effectively stores Cl_2_ and LiCl molecules with enhanced stability. To ascertain the significance of the Al_2_O_3_ group, Li‐Cl_2_ cells were constructed using rGO devoid of this group as a comparative benchmark. As shown in Figure 3a, the initial specific capacity of the Li‐Cl_2_ batteries utilizing rGO was 2000 mAh g^−1^, which declined to 1500 mAh g^−1^ after 60 cycles. This degradation indicates that the absence of the Al_2_O_3_ group, which is crucial for capturing Cl_2_ and LiCl, leads to an insufficient supply of Cl_2_ and suboptimal conversion of LiCl/Cl_2_. To further investigate the significance of the Al_2_O_3_ group, the commercial material Ketjenblack was integrated into Li‐Cl_2_ batteries for comparative analysis. As shown in Figure S13 (Supporting Information), despite Ketjenblack high BET specific surface area of 1400 m^2^ g^−1^, the initial discharge specific capacity was 1200 mAh g^−1^ at the first cycle. However, the CE plummeted to 50% after 50 cycles at a current density of 500 mA g^−1^, with the discharge specific capacity dwindling to only 600 mAh g^−1^. This decline suggests an insufficient supply of Cl_2_ during the redox process, attributable to the weak interaction between Cl_2_ and carbon. It is worth mentioning that when the Li metal anode was replaced with a Na metal anode, the Na‐Cl_2_@Al_2_O_3_@rGO cell exhibited a cycle life close to 120 cycles at a charge capacity of 2500 mAh g^−1^, with a CE of ≈100% (Figure S14, Supporting Information). Figure S15 (Supporting Information) shows the SEM image of the cathode from Li‐Cl_2_ battery after approximately 180 cycles. It is also mentioned that the cathode structure remains stable without significant structural degradation, indicating good long‐term cycling and structural stability of the Al_2_O_3_@rGO cathode when compared to the fresh cathode without cycling as shown in Figure S9 (Supporting Information). This outcome underscores the versatility of Al_2_O_3_@rGO when applied in various alkali metal‐Cl_2_ batteries, and highlights its potential in the realm of rechargeable Cl‐based batteries.

To assess the cycling performance of Al_2_O_3_@rGO in Li‐Cl_2_ batteries across various charging capacities, Li‐Cl_2_@Al_2_O_3_@rGO batteries were subjected to electrochemical testing within a charging capacity range of 700 to 5000 mAh g^−1^. As shown in Figure 3e, the CE remained essentially at 100% as the capacity was ramped up from 700 to 5000 mAh g^−1^. Remarkably, even at the exceedingly high capacity of 5000 mAh g^−1^, the CE could still approach 100%. This demonstrates the exceptional adsorptive and storage capabilities of Al_2_O_3_@rGO for Cl_2_ gas and LiCl, underscoring its effectiveness in high‐performance Li‐Cl_2_ battery applications. Figure 3f illustrates the distribution of charge and discharge voltage profiles for various charge capacities at a current density of 800 mA g^−1^. It is noteworthy that the Li‐Cl_2_@Al_2_O_3_@rGO battery exhibits an overpotential was merely 0.23 V, with a charge voltage of ≈3.82 V and a discharge voltage of ≈3.59 V. To delve into the reaction kinetics of the Li‐Cl_2_@Al_2_O_3_@rGO cell, its cycling performance at different current densities was evaluated as shown in Figure S16 (Supporting Information). When the charge capacity was fixed at 1500 mAh g^−1^ and the current density was escalated from 300 to 2000 mA g^−1^, the distribution of the charging and discharging voltage curves was observed. The discharge voltage plateau decreased from 3.5 to 3.4 V, indicating that Al_2_O_3_@rGO can rapidly supply sufficient Cl_2_ gas, facilitating the swift reduction of Cl_2_ to LiCl. Concurrently, the charging voltage escalated from 3.88 to 3.97 V as the current density was ramped up from 300 to 2000 mA g^−1^ (Figure S16, Supporting Information). This increment suggests that the oxidation kinetics of the conversion from LiCl to Cl_2_ are also swift, likely due to the robust binding effect of LiCl to Al_2_O_3_@rGO. As shown in Figure S16 (Supporting Information), when the charging capacity was set at 1500 mAh g^−1^, the Li‐Cl_2_@Al_2_O_3_@rGO battery maintained a specific capacity of ≈1500 mAh g^−1^ across a range of current densities from 300 to 2000 mA g^−1^. It is noteworthy that the CE is higher than 100% at current densities below 2000 mA g^−1^. It is surmised that this phenomenon might be attributed to the large specific surface area and high porosity of rGO, which allows for additional SOCl_2_ decomposition to participate in the electrochemical reactions at each cycle. Galvanostatic intermittent titration technique (GITT) experiments were conducted to determine the ion diffusion coefficients and to analyze the electrode interface kinetics to guide the electrode materials design. Figure S17 (Supporting Information) presents the GITT curves of rGO and Al_2_O_3_@rGO, revealing that rGO exhibits smaller average voltage polarization compared to Al_2_O_3_@rGO. This phenomenon can be attributed to the strong chlorine adsorption capability of the ultrathin amorphous Al_2_O_3_ layer deposited via ALD technology, which contributes to the higher specific capacity of our Li‐Cl_2_ batteries. However, the disordered atomic arrangement of the amorphous Al_2_O_3_ coating partially hinders electron transport. The lithium‐ion diffusion coefficients for both materials were calculated by applying Fick's second law. The figures illustrate the evolution of lithium‐ion diffusion coefficients during the charge/discharge processes. The results demonstrate that Al_2_O_3_@rGO achieves superior ion diffusion coefficients compared to rGO, likely due to two synergistic effects. The amorphous Al_2_O_3_ coating formed by ALD technology establishes a stable interfacial layer, effectively suppressing side reactions between the electrode and electrolyte. The disordered and polar interface of the amorphous Al_2_O_3_ layer provides additional ion migration pathways, thereby enhancing overall ion transport efficiency.

When the battery was charged at a fixed specific capacity of 1500 mAh g^−1^, the CE remained at ≈100%, even at a high current density of 2000 mA g^−1^. Delightfully, the Li‐Cl_2_@Al_2_O_3_@rGO cells outperform existing reported Li‐Cl_2_ batteries assembled with porous carbon materials in terms of accumulated specific capacity, CE, and maximal discharge capacity, as illustrated in Figure 3d. Notably, the Li‐Cl_2_@Al_2_O_3_@rGO battery boasts a high cumulative specific capacity of up to 440 000 mAh g^−1^, which is approximately 5 times higher than that of the reported porous Li‐Cl_2_@Al_2_O_3_@rGO batteries. Moreover, the applied cycling current density can be elevated from 300 to 2000 mA g^−1^, marking a 13‐fold increase over that of the reported porous carbon Li‐Cl_2_ cells.^[^ ^15^ ^]^ Additionally, the operating temperature range of our Li‐Cl_2_@Al_2_O_3_@rGO battery has been expanded from ‐40 °C to 25 °C, making it suitable for a broader array of applications. These distinctive advantages indicate that Al_2_O_3_@rGO is a highly promising cathode material for high‐performance Li‐Cl_2_ batteries.

**Figure 4:**
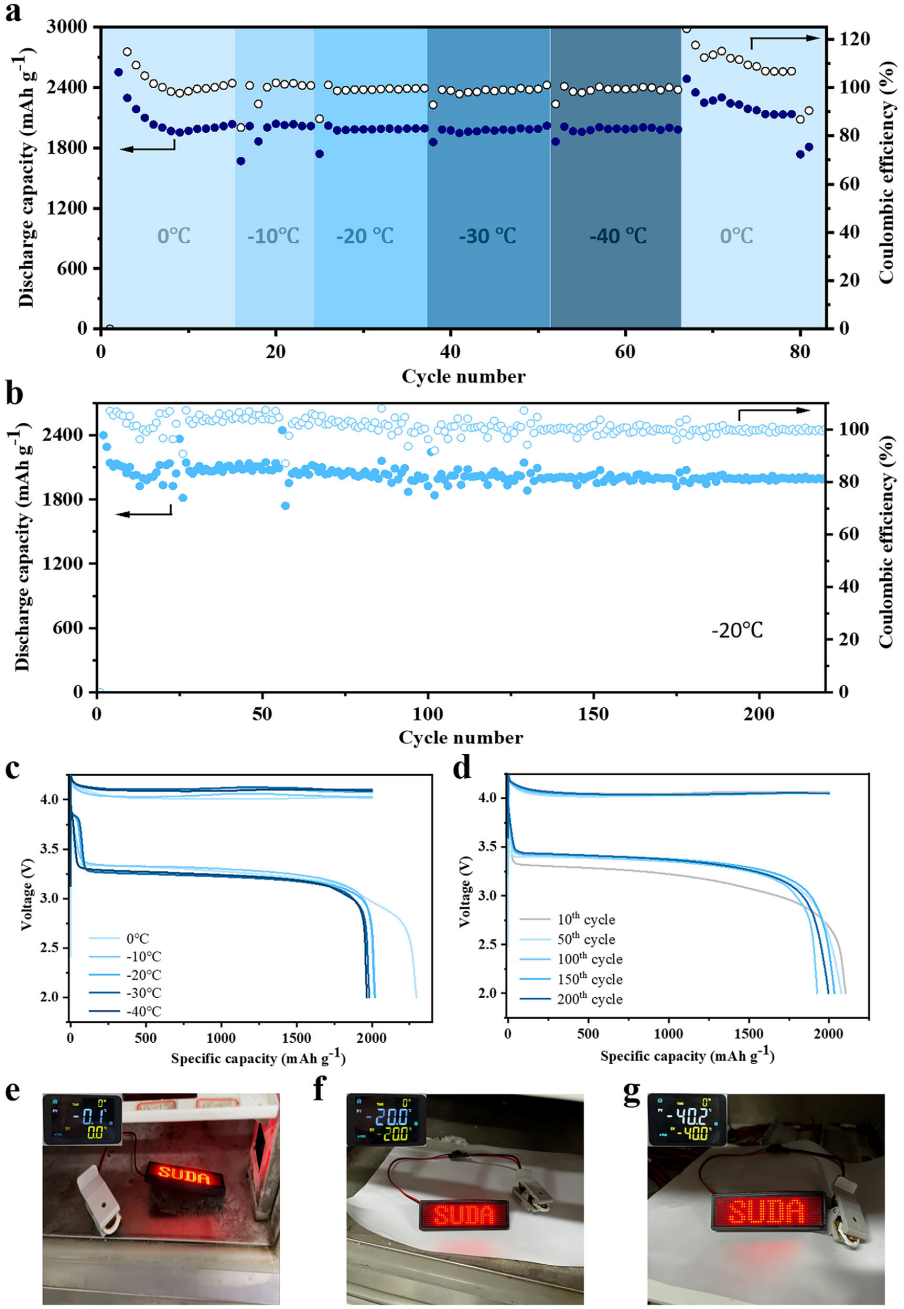
Electrochemical performances of the Li‐Cl_2_@Al_2_O_3_@rGO cell at low temperatures. a) Cycling performance of the Li‐Cl_2_@Al_2_O_3_@rGO cell from 0 to ‐40 °C. b) Cycling performance of the Li‐Cl_2_@Al_2_O_3_@rGO cell at ‐20 °C under charge capacities of 2000 mAh g^−1^. c) Voltage profiles of the Li‐Cl_2_@Al_2_O_3_@rGO cell from 0 to ‐40 °C. d) Voltage profiles of the Li‐Cl_2_@Al_2_O_3_@rGO cell at ‐20 °C at different cycles under charge capacities of 2000 mAh g^−1^. Digital photos of the LED illuminated by the Li‐Cl_2_@Al_2_O_3_@rGO coin cell at low temperatures of e) 0 °C, f) ‐20 °C, and g) ‐40 °C.

The low‐temperature performance of energy storage devices is crucial, especially in cold regions. To assess the potential of Li‐Cl_2_@Al_2_O_3_@rGO batteries for use in cold weather conditions, their electrochemical performances at low temperatures were evaluated. **Figure** **4**a delineates the electrochemical performances of the Li‐Cl_2_@ Al_2_O_3_@rGO battery across a temperature gradient from 0 °C to ‐40 °C. At 0 °C, with a charge capacity of 2000 mAh g^−1^, the discharge capacity of the Li‐Cl_2_@Al_2_O_3_@rGO battery reaches ≈2100 mAh g^−1^. When the temperature is reduced to ‐10 °C, the Li‐Cl_2_@Al_2_O_3_@rGO battery still delivers a substantial discharge capacity of 2000 mAh g^−1^, demonstrating its robust performance even in frigid conditions. When the temperature was further lowered to ‐40 °C, the Li‐Cl_2_@Al_2_O_3_@rGO battery continued to deliver a discharge capacity of ≈2000 mAh g^−1^. At temperatures of 0 °C, ‐10 °C, ‐20 °C, ‐30 °C, and ‐40 °C, the CE can be maintained at ≈105%, ≈100%, ≈99.7%, ≈99.3%, and ≈98.5%, respectively. These impressive electrochemical performances indicate that the Cl_2_ gas supply remains sufficient even at low temperatures, and that the reversibility between Cl_2_ and LiCl is well‐maintained. Moreover, when the temperature was increased from ‐40 °C to 0 °C, the discharge capacity remained stable at ≈2000 mAh g^−1^, demonstrating that the Li‐Cl_2_@Al_2_O_3_@rGO battery exhibits excellent low‐temperature tolerance. It is noteworthy that the CE exceeds 100% when the temperature is above ‐30 °C, which may be attributed to the large specific surface area and high porosity of Al_2_O_3_@rGO, lead to additional SOCl_2_ decomposition in the electrolyte that participates in the reaction. The discharge plateau of the Li‐Cl_2_@Al_2_O_3_@rGO battery is 3.33 V at 0 °C in Figure 4c, and when the temperature drops to ‐30 °C, the discharge plateau is 3.25 V, a decrease of 0.08 V compared to that at 0 °C. This indicates that the reaction kinetics for the conversion of Cl_2_ to LiCl is fast even at low temperatures, further demonstrating the strong bonding between Cl_2_ gas and Al_2_O_3_. It is also worth mentioning that the polarization rate during charge is higher than that during discharge as the temperature decreases, suggests that the effect of temperature on the solid‐gas reaction kinetics of LiCl to Cl_2_ gas is more significant than that of the gas‐solid reaction kinetics of Cl_2_ gas to LiCl. In addition to the excellent low‐temperature performance, we also tested the long‐term cycling stability of the Li‐Cl_2_@Al_2_O_3_@rGO battery at ‐20 °C. The Li‐Cl_2_@Al_2_O_3_@rGO battery exhibits an impressive initial discharge capacity of 9570 mAh g^−1^ at ‐20 °C in Figure S18 (Supporting Information) and maintains a high specific capacity of 2000 mAh g^−1^ with a charge capacity of 2000 mAh g^−1^, along with a cycle life over 200 cycles and a capacity retention rate of ≈99.8% in Figure 4b. Moreover, the Li‐Cl_2_@ Al_2_O_3_@rGO battery sustains a discharge voltage plateau at 3.4 V over more than 200 cycles (Figure 4d), which attests to its enduring performance at low temperatures. We further utilized a Li‐Cl_2_@Al_2_O_3_@rGO coin cell to light up an LED display at different temperature of 0 °C, −20 °C, and −40 °C, as shown in Figure 4e–g. This demonstration confirms that the Li‐Cl_2_@Al_2_O_3_@rGO coin cell can reliably supply power to the LED display even at the low temperature of −40 °C, confirmed its practicability in cold conditions.

**Figure 5:**
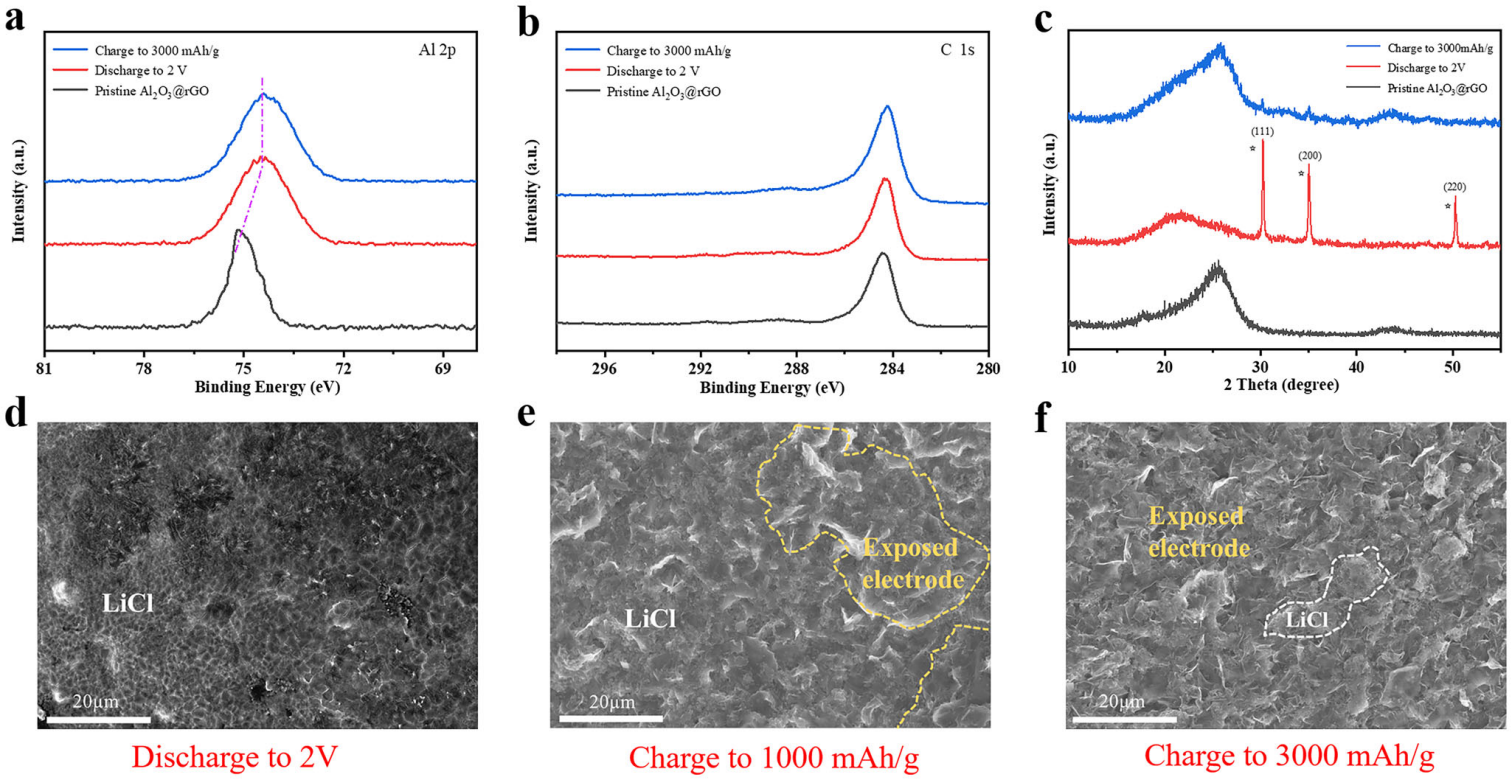
Enrichment mechanism of Cl_2_ by Al_2_O_3_@rGO and dynamic respiratory interfaces under electrochemical conditions. a) Al 2p and b) C 1s XPS spectra of pristine Al_2_O_3_@rGO, Al_2_O_3_@rGO discharged to 2 V, and Al_2_O_3_@rGO charged to 3000 mAh g^−1^. c) XRD of Al_2_O_3_@rGO at the states of pristine Al_2_O_3_@rGO, discharged to 2 V and charged to 3000 mAh g^−1^. SEM images of Al_2_O_3_@rGO at the states of d) discharged to 2 V, e) charged to 1000 mAh g^−1^, and f) charged to 3000 mAh g^−1^.

Moreover, multiple techniques such as XPS, XRD and SEM were further employed to elucidate the underlying mechanism of Cl_2_ enrichment in Al_2_O_3_@rGO under electrochemical conditions. Initially, the cathodes were subjected to the XPS analysis under three distinct electrochemical states: pristine Al_2_O_3_@rGO, Al_2_O_3_@rGO discharged to 2 V, and Al_2_O_3_@rGO charged to 3000 mAh g^−1^. As shown in **Figure** **5**a, the Al 2p XPS peak, centered at ≈75.1 eV, is characteristic of Al_2_O_3_ in the pristine Al_2_O_3_@rGO electrode. This peak shifts to ≈74.5 eV upon discharging the Al_2_O_3_@rGO electrode to 2 V and charging it to 3000 mAh g^−1^. The shift towards lower energy indicates a transfer of electrons from Al atoms to Cl atoms, likely due to the adsorption of Cl_2_ / LiCl. These XPS findings are broadly consistent with our computational predictions. Furthermore, the C 1s XPS spectrum reveals an unchanging peak across various charging and discharging states (Figure 5b), which underscores the robustness of the Al_2_O_3_@rGO material under electrochemical conditions. As shown in Figure 5c when the cathodes were subjected to the XRD analysis, the crystal surfaces of LiCl (111), LiCl (200), and LiCl (220) are distinctly observable during the discharge to 2 V. In contrast, the diffraction peaks associated with LiCl are nearly absent upon charging to 3000 mAh g^−1^. The Al_2_O_3_@rGO cathode was subjected to further SEM analysis across three distinct states: discharge to 2 V, charge to 1000 mAh g^−1^, and charge to 3000 mAh g^−1^. As shown in Figure 5d, upon discharge to 2 V, the surface of the Al_2_O_3_@rGO electrode was nearly entirely obscured by LiCl. With an increase in charging depth from 1000 mAh g^−1^ (Figure 5e) to 3000 mAh g^−1^ (Figure 5f), the exposed surface area of the Al_2_O_3_@rGO electrode progressively expands. This observation suggests that the LiCl deposited on the Al_2_O_3_@rGO surface is highly reversible, and as the charging depth escalates, a greater amount of LiCl is converted into Cl_2_ gas. A stable dynamic Cl_2_/LiCl respiratory mechanism mediated by ALD Al_2_O_3_ conformal metasurface exists at the solid‐liquid‐gas three‐phase interfaces of the cathode. The atomic layer of Al_2_O_3_ optimized interface characteristics of the ultrahigh‐capacity cathode, thereby increasing the number of electrocatalytic active sites and boosting the reaction rate.^[^ ^39^ ^]^ In conclusion, Cl_2_ gas readily engages in chemical interactions with Al_2_O_3_ groups, ensuring that Cl_2_ molecules bound to Al_2_O_3_ are continuously and swiftly available for the reduction reaction that generates LiCl during the discharge process, leading to an elevated discharge capacity. The LiCl produced is stored within the pores of the rGO. During the charging phase, the newly formed Cl_2_ is effectively captured by the Al_2_O_3_ groups, thereby mitigating its shuttle effect. The synergistic interaction between rGO and Al_2_O_3_ ensures a prolonged cycle life for Li‐Cl_2_ batteries at high discharge capacities by effective adsorption and fast kinetics, and stable dynamic respiratory interfaces.

## 3 Conclusion

In summary, we have engineered an Al_2_O_3_@rGO material replete with abundant binding sites, rendering it an ideal ultrahigh‐capacity cathode material for rechargeable Li‐Cl_2_ batteries. Its highly stable hierarchical starburst porous structure by the microemulsion skin effect and subsequently deposited Al_2_O_3_ groups by conformal FBALD are pivotal in enhancing the electrochemical performance of these batteries. DFT calculations, corroborated by experimental outcomes, have verified that the Al_2_O_3_ groups effectively enrich Cl_2_ molecules and that the stable architecture of Al_2_O_3_@rGO is conducive to the enrichment of Cl_2_ gas. Owing to the distinctive structure of Al_2_O_3_@rGO, the discharge capacity of Li‐Cl_2_ batteries has been markedly escalated to 5000 mAh g^−1^, with the CE consistently maintained at ≈100%. This performance surpasses that of the current state‐of‐the‐art cathode materials for Li‐Cl_2_ batteries. Furthermore, the Li‐Cl_2_@Al_2_O_3_@rGO battery boasts a high specific capacity of 2500 mAh g^−1^ at room temperature with a cycle life of ≈180 cycles. It also demonstrates outstanding electrochemical performance at a low temperature of ‐20 °C, maintaining a CE of 99.7% at a specific capacity of 2000 mAh g^−1^, featuring a discharge plateau close to 3.5 V and a stable cycle life ≈200 cycles. After the deposition of Al_2_O_3_ by FBALD, both the adsorption capture of Cl_2_ molecules and the catalytic property of LiCl by electrode materials are increased, while the surface deposited Al_2_O_3_ does not affect the excellent electrical and thermal conductivity of rGO, so that a high‐performance Li‐Cl_2_ battery with stable output is obtained. This research paves a new path for the development of cathode materials for rechargeable Li‐Cl_2_ batteries that offer ultrahigh capacity and excellent stability across a broad operating temperature range. The scalable heterostructure design of this hierarchical starburst porous functionalized cathode material with Al_2_O_3_‐skinned conformal metasurfaces expands the repertoire of cathode materials for achieving high‐performance Li‐Cl_2_ battery systems with the features of effective adsorption and fast kinetics, stable dynamic respiratory interface, intelligent thermal management and safe operation over a wide temperature range. However, the exploration of rechargeable alkali metal‐chlorine batteries remains in a relatively nascent stage for sustainable energy storage, and further optimization is necessary to enhance the actual energy density for broader application prospects.

## 4 Experimental Section

### Materials

Graphene oxide (GO) was acquired from Alab (Shanghai) Chemical Technology Co., Ltd. Polytetrafluoroethylene preparation (PTFE, 60 wt%) was obtained from Shanghai Macklin Biochemical Technology Co., Ltd. Commercial carbon cloth (CC) was purchased from Taiwan CeTech Corp., China. Thionyl chloride (SOCl_2_), lithium chloride (LiCl), lithium bis(fluorosulfonyl)imide (LiFSI), and lithium bis(trifluoromethanesulfonyl)imide (LiTFSI) were acquired from Shanghai Aladdin Biochemical Technology Co., Ltd. All reagents used in the experiments were of analytical grade and used without further purification. Deionized (DI) water was used throughout the experiments.

### Synthesis of Starburst Porous Graphene (Starburst rGO)

Starburst porous graphene membranes were crafted from functionalized graphene flakes using the colloidal microemulsion method and the skin effect. The functionalized graphene flakes were derived from the thermal reduction of graphene oxide flakes at 1050 °C for a minute under an argon atmosphere. Subsequently, these functionalized graphene flakes were dispersed in a microemulsion solution containing a binder material (PTFE, 60 wt% solids). The mixture was stirred for 15 min, followed by ultrasonic mixing for 20 min, with these steps being repeated three times to ensure thorough mixing. After the casting and drying processes, a functionalized graphene membrane with a graded porous structure was successfully obtained.

### Preparation of ALD Conformal Starburst Porous Electrodes (Al_2_O_3_@rGO)

ALD Al_2_O_3_‐skinned conformal metasurface heterostructured electrodes were fabricated by the PTFE microemulsion skin effect and subsequent atomic layer epitaxy of the conformal Al_2_O_3_ layer on starburst porous graphene surface. As comparisons, three meta‐structured electrode samples (pristine‐rGO, starburst‐rGO, and Al_2_O_3_@rGO) with anisotropy and degrees of freedom as well as conformal metasurface were fabricated as follows: 1) As shown in Figure S5a (Supporting Information), the initial reduced graphene oxide, Ketjenblack, and PVDF were dispersed in NMP at a mass ratio of 8:1:1 and continuously stirred for 5 h. The resulting slurry was then coated onto a carbon cloth and dried at 60 °C. 2) As shown in Figure S5b (Supporting Information), the rGO powder obtained by the colloidal microemulsion method, Ketjenblack, and PVDF were dispersed in NMP with a weight ratio of 8:1:1 and continuously stirred for 5 h to achieve a homogeneous mixture. The resulting slurry was then cast onto a carbon cloth and dried at 60 °C. 3) As shown in Figure S5c (Supporting Information), the rGO powder obtained by the colloidal microemulsion method, Ketjenblack, and PVDF were dispersed in NMP with a weight ratio of 8:1:1 and continuously stirred for 5 h to achieve a homogeneous mixture. The resulting slurry was then cast onto a carbon cloth and dried at 60 °C. The dried electrodes were subsequently coated with a layer of Al_2_O_3_ groups using the FBALD technique, yielding the functionalized porous electrodes with graphene underlying ALD Al_2_O_3_ dielectrics. O_3_ pretreatment, immediately followed by the ALD process with trimethylaluminum (TMA) /O_3_ chemistry, formed conformal Al_2_O_3_ layers without any preferential deposition at the step edges of graphene, presenting a facile route which combine the functionalization of a starburst porous graphene surface with an ALD process to allow for conformal Al_2_O_3_ layer. The TMA/O_3_ process began to provide nucleation sites on the basal planes of the surface. This is attributed to functionalization of graphene by ozone treatment, imparting a hydrophilic character which is desirable for ALD deposition.

### Assembly of Li‐Cl_2_@Al_2_O_3_@rGO Coin Cells and Electrochemical Measurements

The Li‐Cl_2_ coin cells were assembled from a Li metal anode, measuring 14 mm in diameter and 1 mm in thickness, and a 14 mm diameter Al_2_O_3_@rGO cathode. The electrolyte for Li‐Cl_2_ coin cells was prepared according to the reported literature.^[^ ^15^ ^]^ In summary, 532 mg AlCl_3_, 168 mg LiCl, 88 mg LiTFSI, and 88 mg LiFSI were added into a 20 mL vial, to which 2 mL SOCl_2_ was added and stirred for 30 min, leading to the formation of a pale yellow solution. Three layers of glass fibers were employed as a separator between the cathode and anode, which were then saturated with 100 µL of the prepared electrolyte. The entire cell assembly process was conducted within an Ar‐filled glovebox, where the levels of water and oxygen were maintained below 0.1 ppm. The Li‐Cl_2_ coin cells with Starburst‐rGO or Pristine‐rGO cathode were prepared using the same procedure but the different cathode for comparison. The Na‐Cl_2_ coin cells with Al_2_O_3_@rGO cathode were also assembled using the same procedure but the use of Na metal anode. The assembled cells underwent long‐term constant current cycling tests and multipurpose performance evaluations at different environmental temperatures (from 25 to ‐40 °C) use a LAND programmable battery test system. All electrochemical tests were conducted using a LAND CT2001A battery test system and an electrochemical workstation (CHI660E) for EIS (electrochemical impedance spectroscopy) measurements with a frequency range from 1 MHz to 0.1 Hz. Galvanostatic charge‐discharge characterization was tested in the 2.0 to 4.2 V voltage range.

### Characterizations

Scanning electron microscopy (SEM) images were taken with Hitachi SU‐8010 analytical SEM (Japan). Transmission electron microscopy (TEM) images were acquired from Thermo Scientific Spectra 300 by using an accelerating voltage of 300 kV. X‐ray diffraction (XRD) data were collected on a Bruker D8 X‐ray diffractometer using Cu Kα radiation (λ = 1.5406 Å). X‐ray photoelectron spectroscopy (XPS) spectra were taken from EXCALAB 250 XI X‐ray photoelectron spectrometer system (Thermo Scientific). The defect densities of the materials were characterized using a Raman spectrometer (Horiba, HR Evolution). The specific surface areas and pore size distributions were analyzed using a Brunauer‐Emmett‐Teller (BET) specific surface area analyzer (Micromeritics, ASAP 2460). The FTA‐1000 drop shape instrument was employed for the contact angle tests and hydrophilicity analysis to simulate and evaluate electrolyte wettability or electrode infiltration. The specific contact angle values were fitted by the test software based on the Young‐Laplace equation. The thermal conductivity and heat dissipation of the electrodes were tested using a FLUKE‐PTi120 infrared camera to simulate and evaluate the prevention of battery thermal runaway at elevated temperatures and the battery low‐temperature insulation behaviors at different low temperatures. The thermal images were analyzed using thermal image analysis software.

### Statistical Analysis

The reduced graphene oxide slurry prepared by the microemulsion method was coated onto carbon cloth and cut into 3×4 cm pieces, followed by atomic layer deposition (ALD) of Al_2_O_3_. The resulting 3×4 cm deposited electrodes were then cut into 14 mm diameter circular discs for infrared thermal imaging and contact angle measurements. XPS data were deconvoluted and analyzed using Avantage software, while XRD data were characterized and processed with Jade9 software. EIS data were recorded by CHI660E software and fitted to model the spectral resistance using ZSimpWin software. All other measured data were plotted as documented.

## Conflict of Interest

The authors declare no conflict of interest.

## Supporting information



Supporting Information

## Data Availability

The data supporting the findings of this study are available from the corresponding author upon reasonable request.
