# Supplementary material for: High‐Performance Rechargeable Lithium‐Chlorine Batteries with ALD Conformal Starburst Porous Graphene Positive Electrodes
Source: Adv Sci (Weinh). 2025 Jun 23;12(30):e03113. doi: 10.1002/advs.202503113 (PMC12376684; doi:10.1002/advs.202503113)
Supplement: Supplementary file 1 — Supporting Information [file ADVS-12-e03113-s001.docx]

Supporting Information

**High-Performance Rechargeable Lithium-Chlorine Batteries with ALD Conformal Starburst Porous Graphene Positive Electrodes**

Zhuo Yang, Yanan Huang, Weicheng Zhou, Hong Fan, Zhihao Ding, Xu Yan, Yu Lu, Alexander S. Sigov, Wei Huang, Lijun Gao, and Cheng Huang*

1. Yang, Y.N. Huang, W.C. Zhou, H. Fan, Prof. L.J. Gao, Prof. C. Huang

Soochow Institute for Energy and Materials InnovationS (SIEMIS)

Key Laboratory of Advanced Carbon Materials and Wearable Energy Technologies of Jiangsu Province

Key Laboratory of Core Technology of High Specific Energy Battery and Key Materials for Petroleum and Chemical Industry

College of Energy, Soochow University, 688 Moye Road, Suzhou 215006, P. R. China

E-mail: [chengh@suda.edu.cn](mailto:chengh@suda.edu.cn)

Z. Yang, Y.N. Huang, W.C. Zhou, Z.H. Ding, X. Yan, Y. Lu, Prof. A.S. Sigov, Prof. C. Huang

Physics and Energy Department, Volta and DiPole Materials Labs

International Joint MetaCenter for Advanced Photonics and Electronics

School of Optical and Electronic Information, Suzhou City University, 1188 Wuzhong District, Suzhou 215006, P. R. China

E-mail: [chengh@szcu.edu.cn](mailto:chengh@suda.edu.cn)

1. Fan, Prof. A.S. Sigov, Prof. C. Huang, Prof. W. Huang

School of Flexible Electronics & State Key Laboratory of Optoelectronic Materials and Technologies

Sun Yat-sen University, 66 Gongchang Road, Guangming District, Shenzhen 518107, P.R. China

Prof. C. Huang

Pacific Northwest National Laboratory, Richland, Washington 99352, USA

Z. Yang, Y.N. Huang, Prof. C. Huang, Prof. W. Huang

Institute of Advanced Materials and Institute of Membrane Science and Technology

Jiangsu National Synergistic Innovation Center for Advanced Materials

State Key Laboratory of Flexible Electronics

Suzhou Laboratory and Nanjing Tech University, Nanjing 211816, P. R. China

**The calculation method**

In this work, all calculations were performed using the Vienna Ab initio Simulation Package (VASP) with the projector augmented wave (PAW) method.^[1-4]^ The Perdew-Burke-Ernzerhof (PBE)^[5]^ functional, which employs the generalized gradient approximation (GGA), was utilized for the exchange-correlation functional, complemented by the DFT-D3 correction.^[6,7]^ The plane-wave basis set had a cut-off energy of 500 eV. A vacuum layer of 15 Å was incorporated into all surface models to prevent interactions between periodic images. The Brillouin zone was sampled using a 3*3*1 Gamma-centered k-points grid for Al_2_O_3_ model and graphene model. Self-consistent calculations were conducted with a convergence energy threshold of 10^-5 eV, and the equilibrium geometries were optimized to ensure that the maximum stress on each atom not exceed 0.02 eV/Å. For the adsorption reactions of molecules, the energies were computed using the following equations:


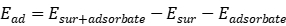


Where
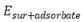
 is the total energy of adsorption model,
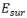
 is the total energy of surface and
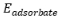
 is the total energy of adsorbate.


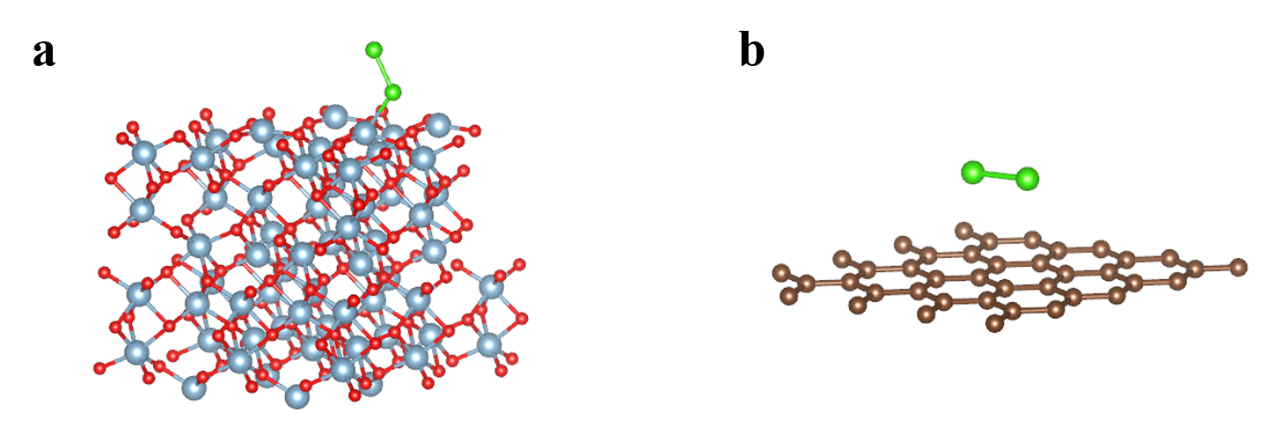


**Figure S1.** Optimized structures of (a) Al_2_O_3_ and (b) graphene with adsorbed Cl_2_.


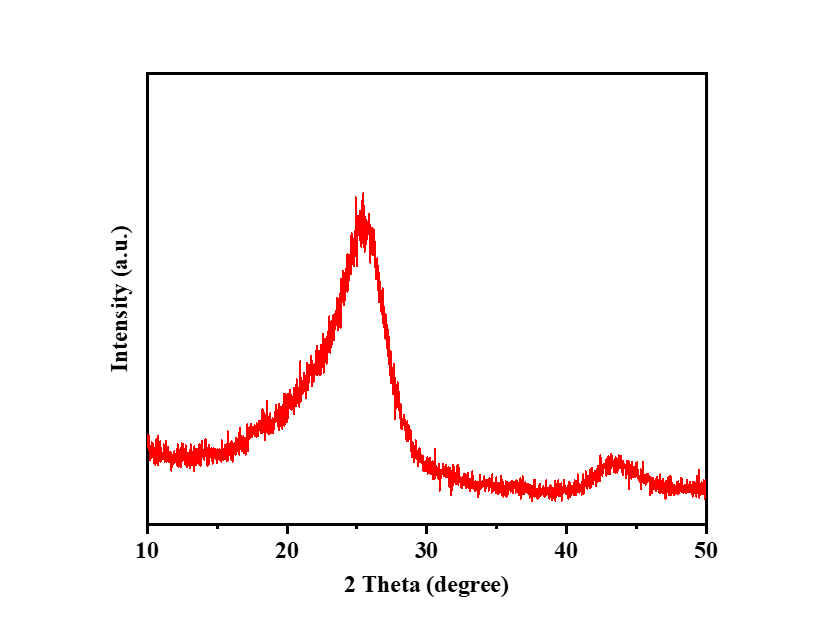


**Figure S2.** XRD of the prepared starburst rGO hierarchical porous electrode by the PTFE microemulsion skin effect (rGO).


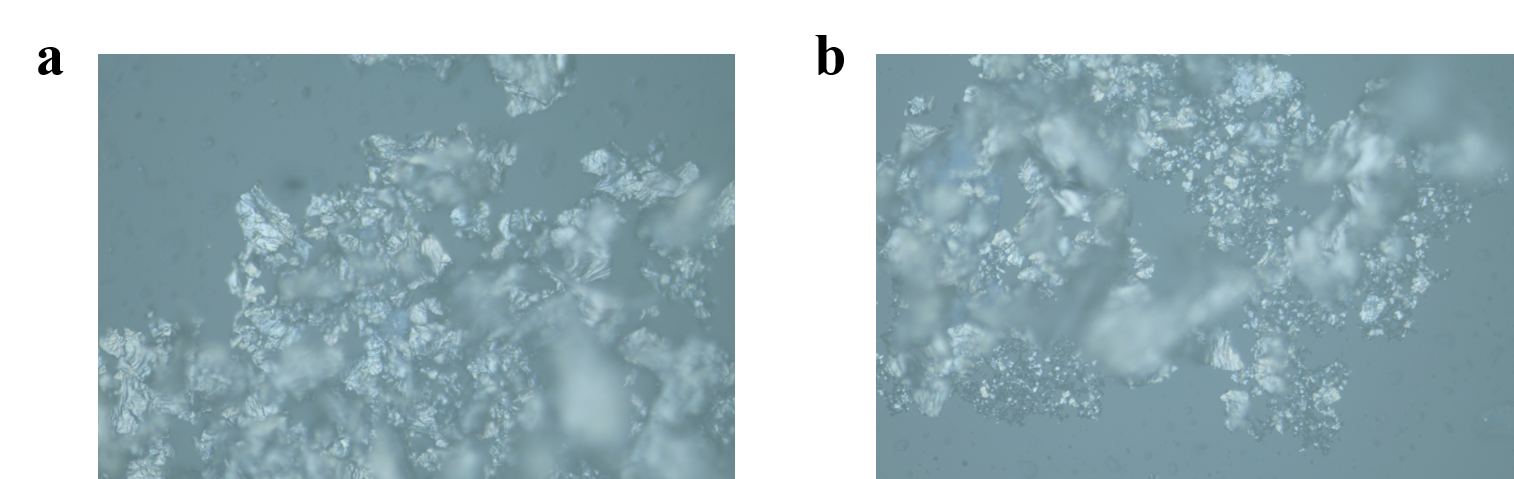


**Figure S3.** The optical images of (a) starburst-rGO and (b) Al_2_O_3_@rGO.


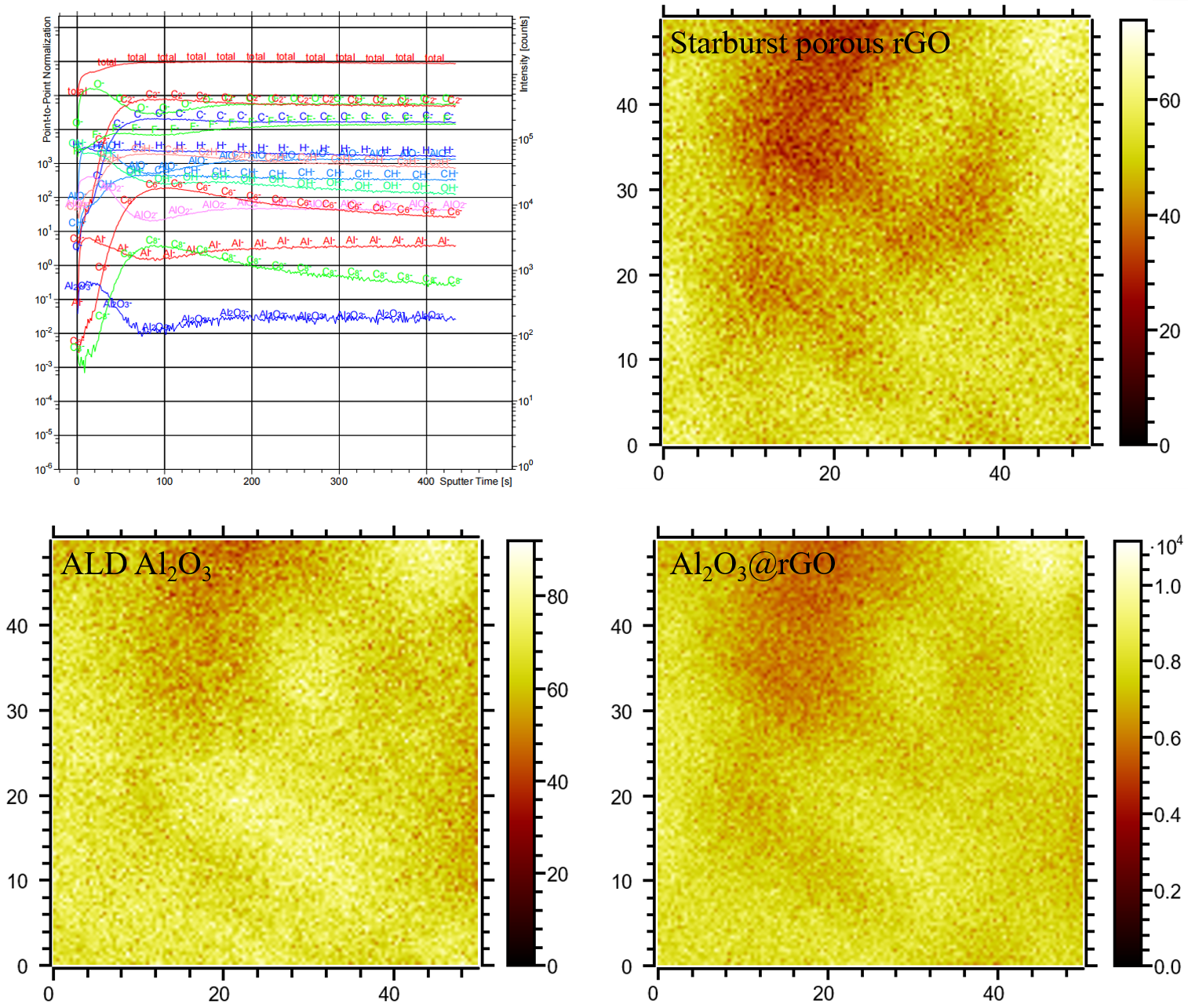


**Figure S4.** TOF-SIMS sputter depth profiles and 2D surface chemical mapping (analysis area: 50 × 50 µm^2^) of the Al_2_O_3_@rGO electrode.


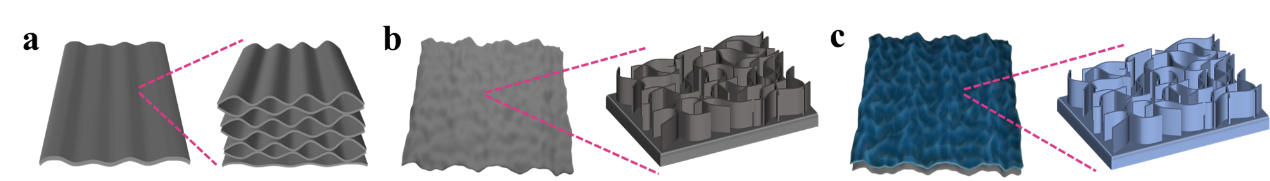


**Figure S5.** The schematic diagrams for structures of these three meta-structured electrode samples with anisotropy and degrees of freedom as well as conformal metasurface: (a) traditional rGO textured electrode with inclined homogeneous or horizontal alignment (Pristine-rGO), (b) starburst rGO hierarchical porous electrode with inclined homeotropic or vertical alignment by the PTFE microemulsion skin effect (Starburst-rGO), and (c) ALD Al_2_O_3_-skinned conformal metasurface heterostructured electrode by the PTFE microemulsion skin effect and subsequent atomic layer epitaxy (Al_2_O_3_@rGO).


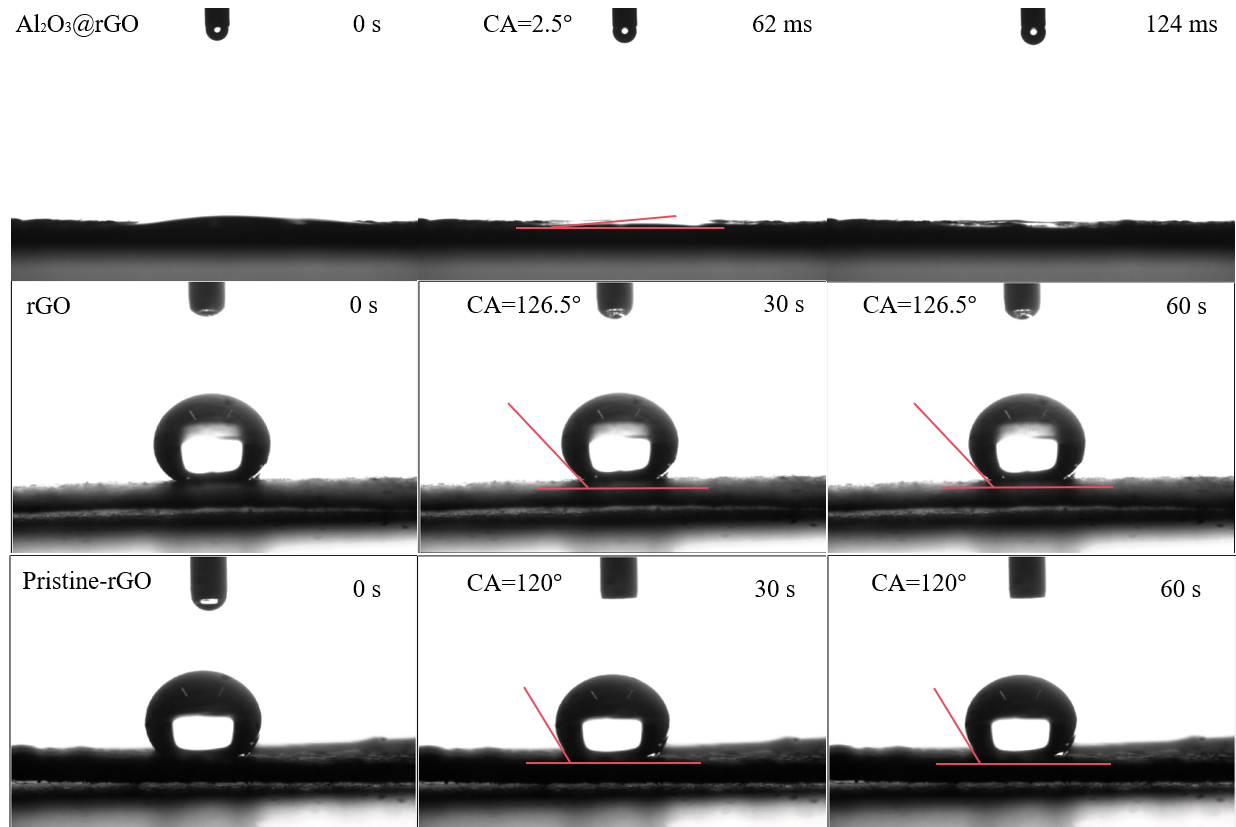


**Figure S6.** The contact angle tests of these three samples to simulate and evaluate electrolyte wettability or infiltration: Pristine-rGO with hydrophobicity, Starburst-rGO with enhanced hydrophobicity, and Al_2_O_3_@rGO with superhydrophilicity.


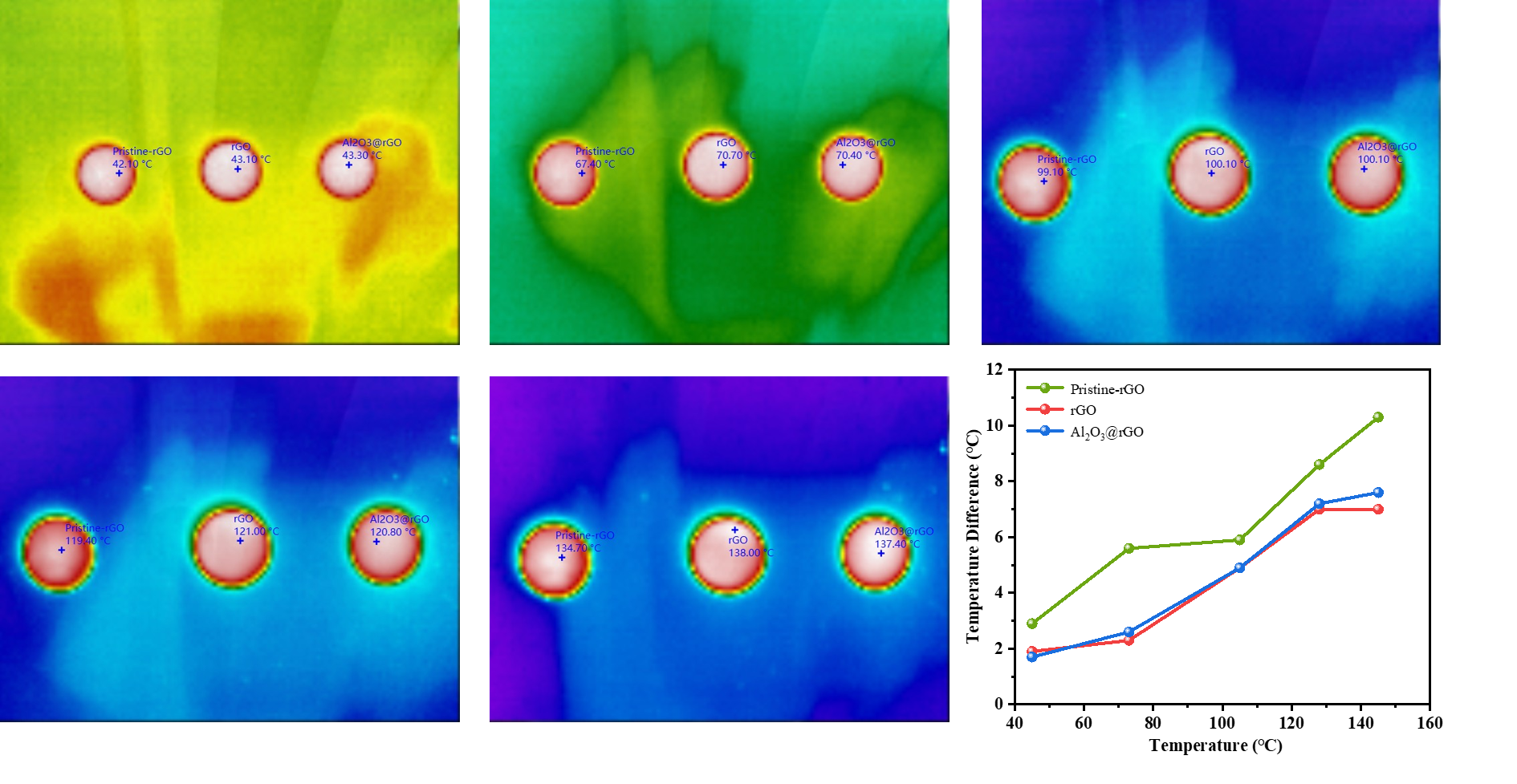


**Figure S7.** The thermal conductivity and heat dissipation tests of these three samples at elevated temperatures to simulate and evaluate the prevention of battery thermal runaway: Starburst-rGO and Al_2_O_3_@rGO are superior to Pristine-rGO due to anisotropic thermal conductivities at the horizontal and vertical level of starburst rGO and Al_2_O_3._


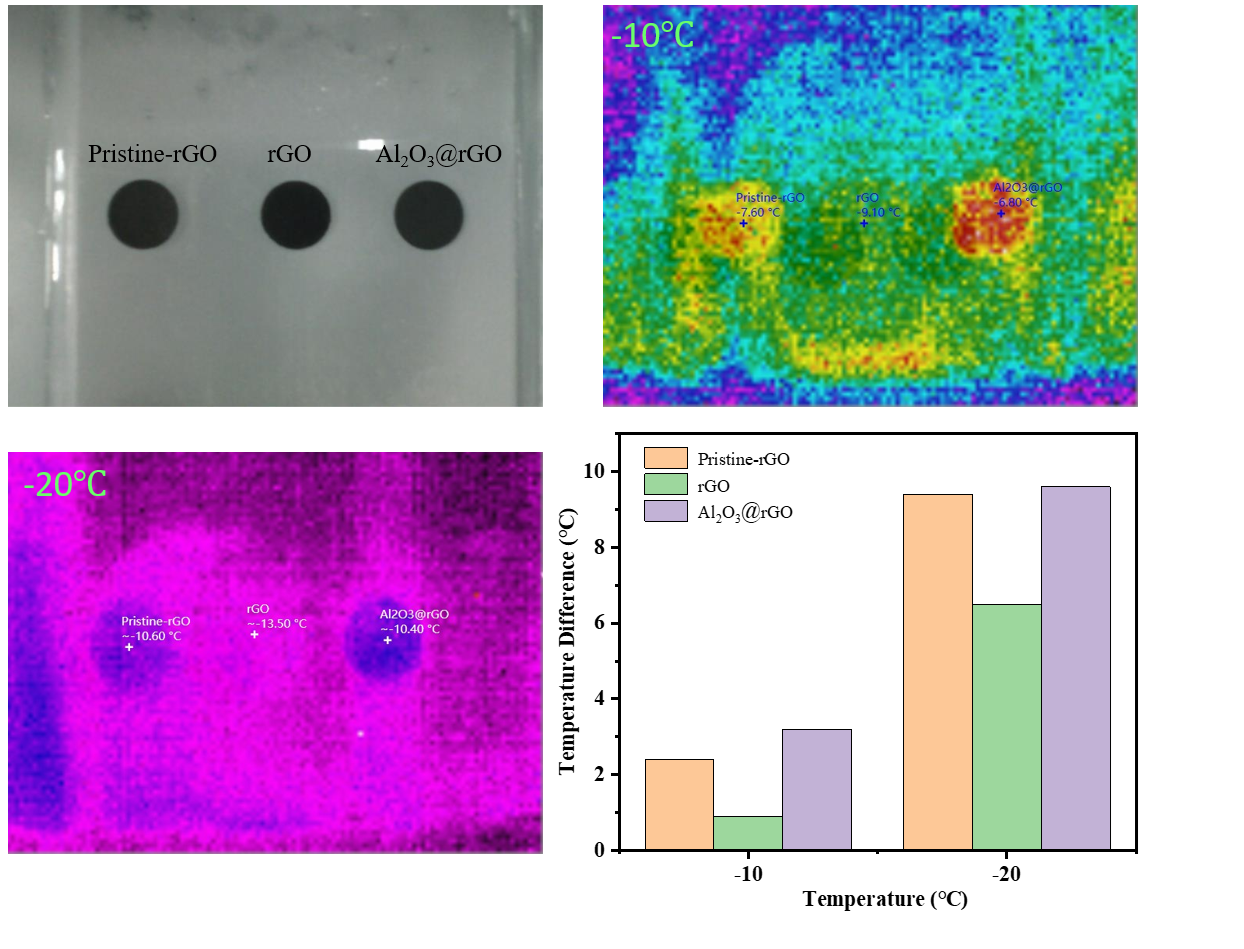


**Figure S8.** The thermal conductivity and heat dissipation tests of these three samples at different low temperatures to simulate and evaluate the battery low-temperature insulation behaviors: Al_2_O_3_@rGO and Pristine-rGO are superior to Starburst-rGO due to anisotropic thermal conductivities at the horizontal and vertical level of Pristine-rGO and Al_2_O_3._

_,_


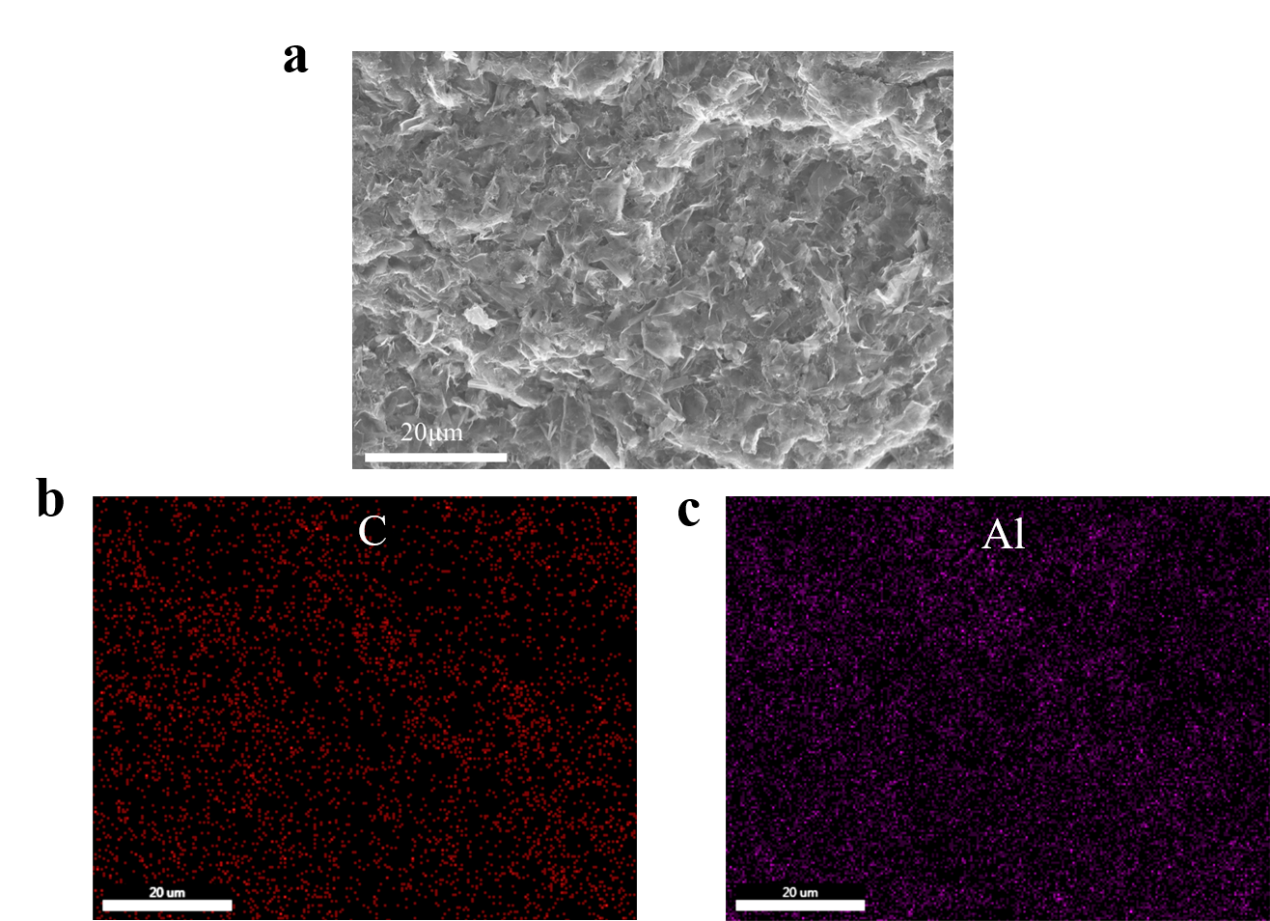


**Figure S9.** The SEM image and corresponding EDX elemental mapping of Al_2_O_3_@rGO electrode.


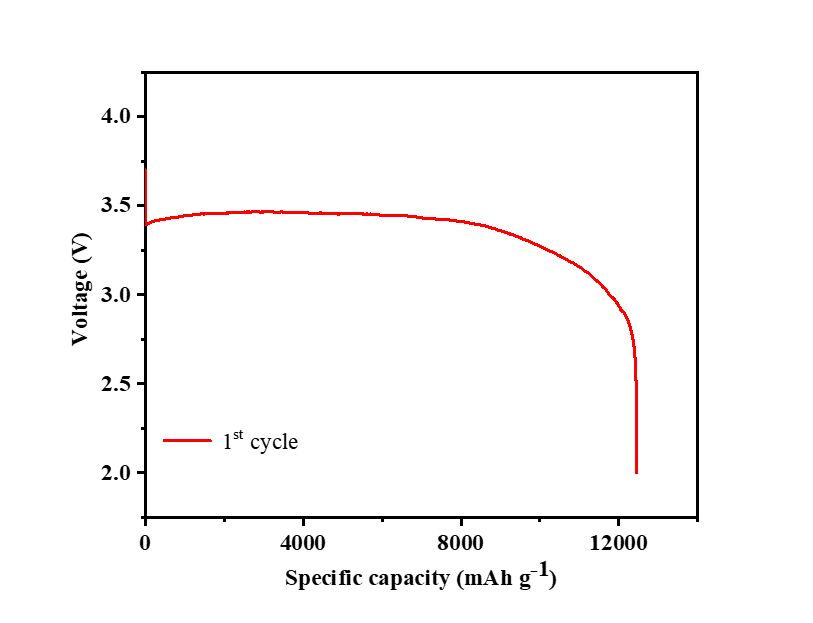


**Figure S10.** The discharge voltage profile of Li-Cl_2_@Al_2_O_3_@rGO cell at the first cycle.


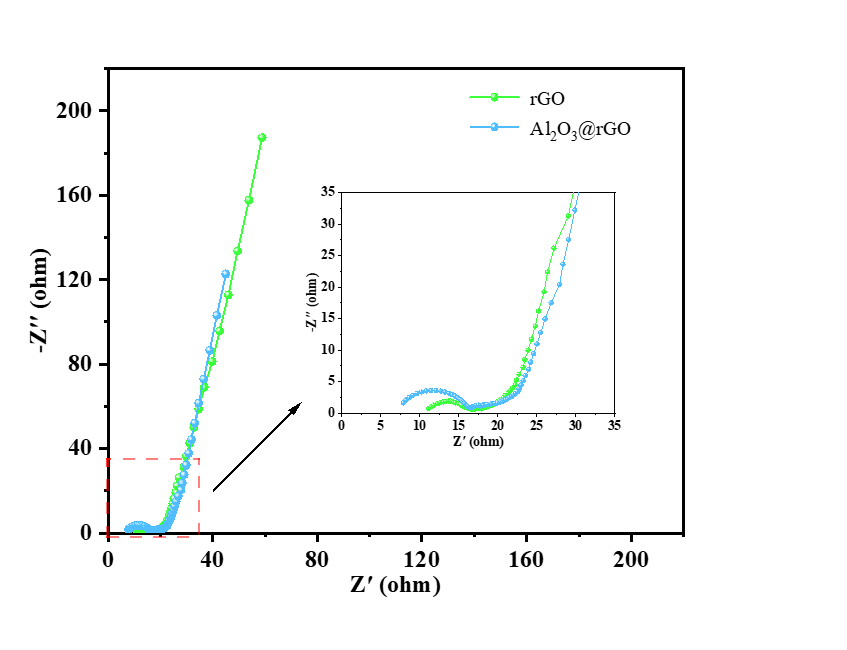


**Figure S11.** EIS curves of Starburst-rGO and Al_2_O_3_@rGO electrodes in the initial state.


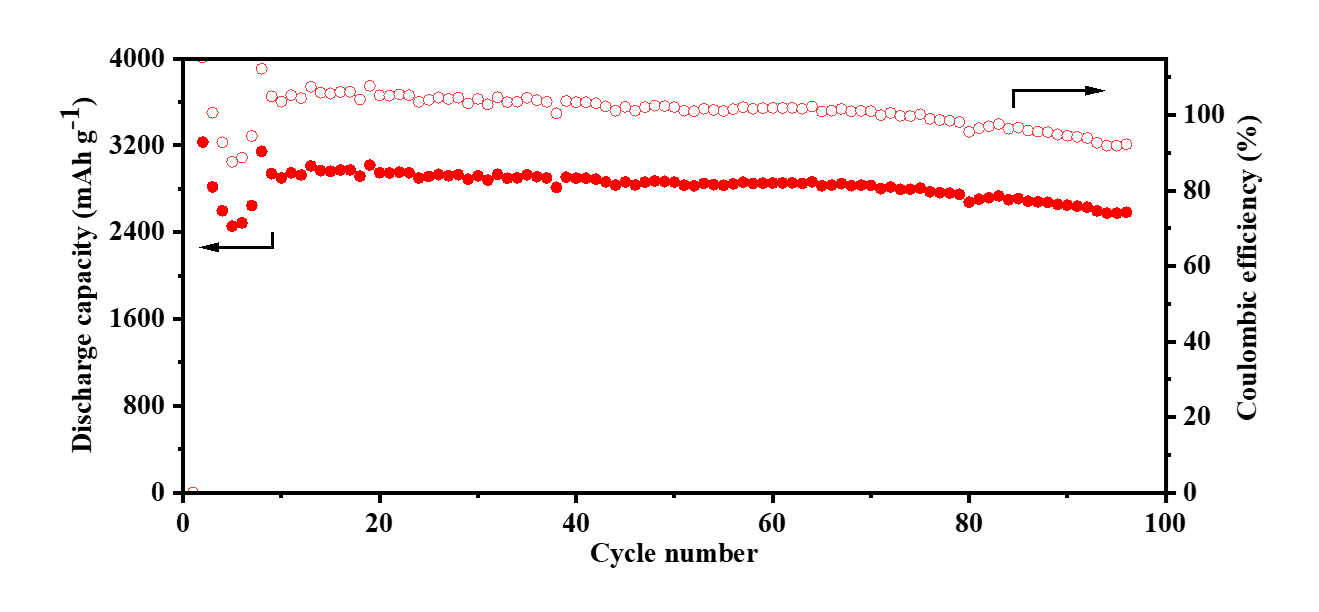


**Figure S12.** The cycling performance of Li-Cl_2_@Al_2_O_3_@rGO cell when setting the charge capacity at 2800 mAh/g.


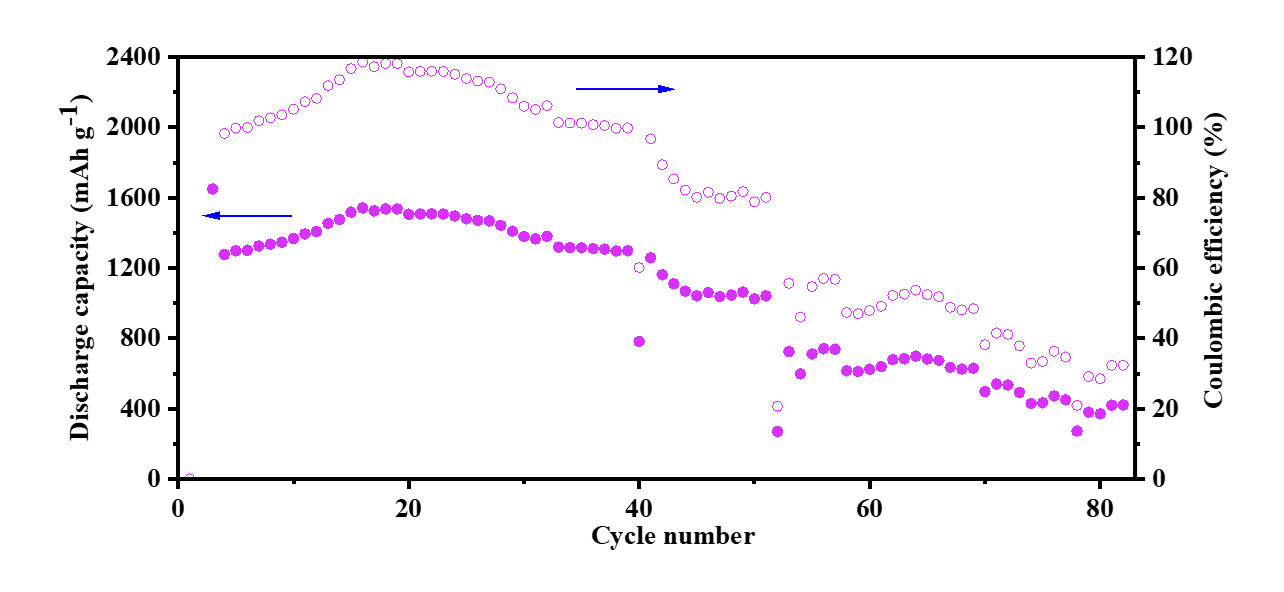


**Figure S13.** The electrochemical performance of Li-Cl_2_@ketjenblack cell. Cycling performance of Li-Cl_2_@ketjenblack cell at a current density of 300 mA/g at room temperature.


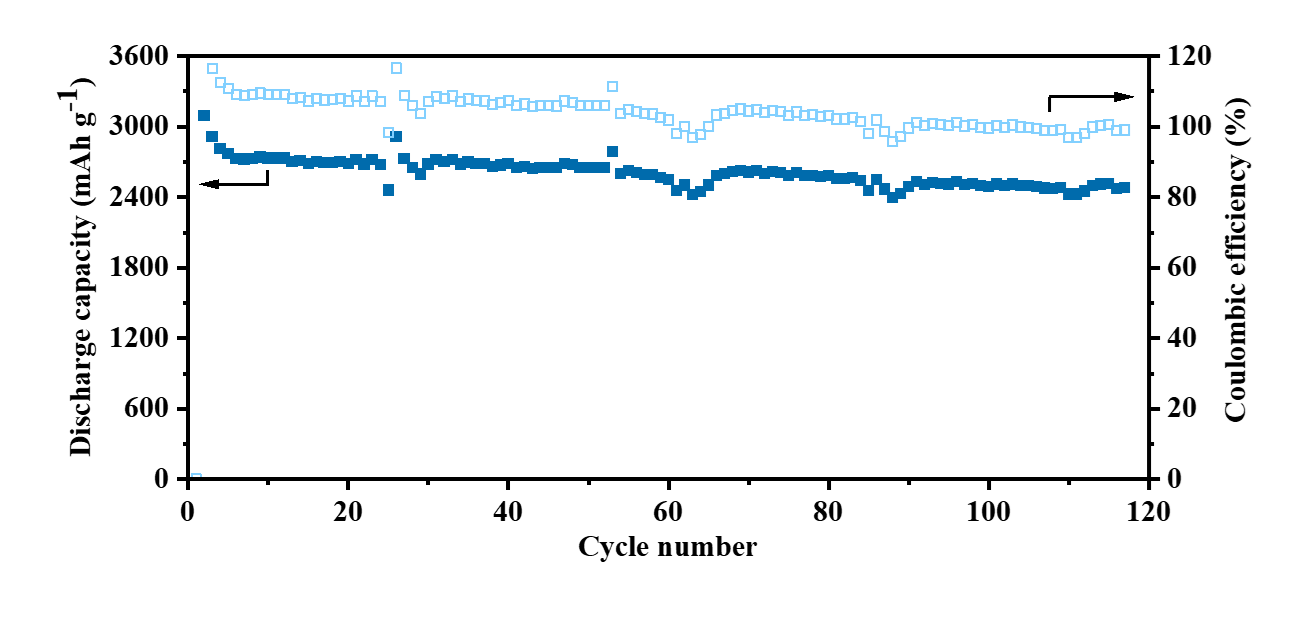


**Figure S14.** The cycling performance of Na-Cl_2_@Al_2_O_3_@rGO cell when setting the charge capacity at 2500 mAh/g.


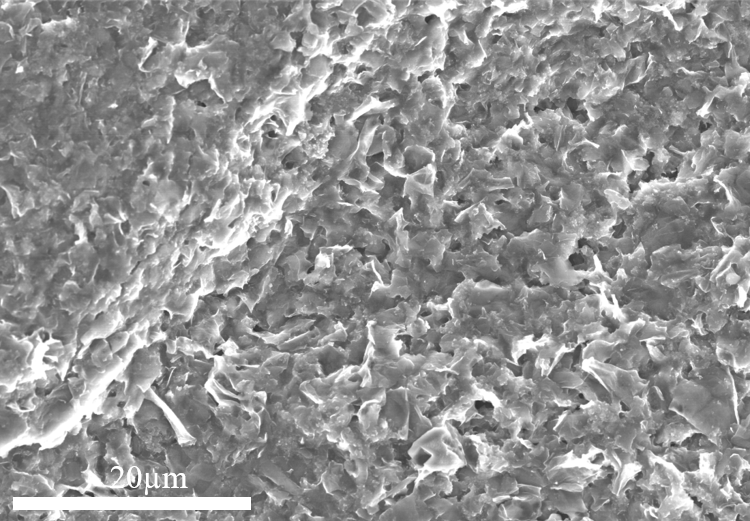


**Figure S15.** The SEM image of the cathode from Li-Cl_2_ battery after approximately 180 cycles.


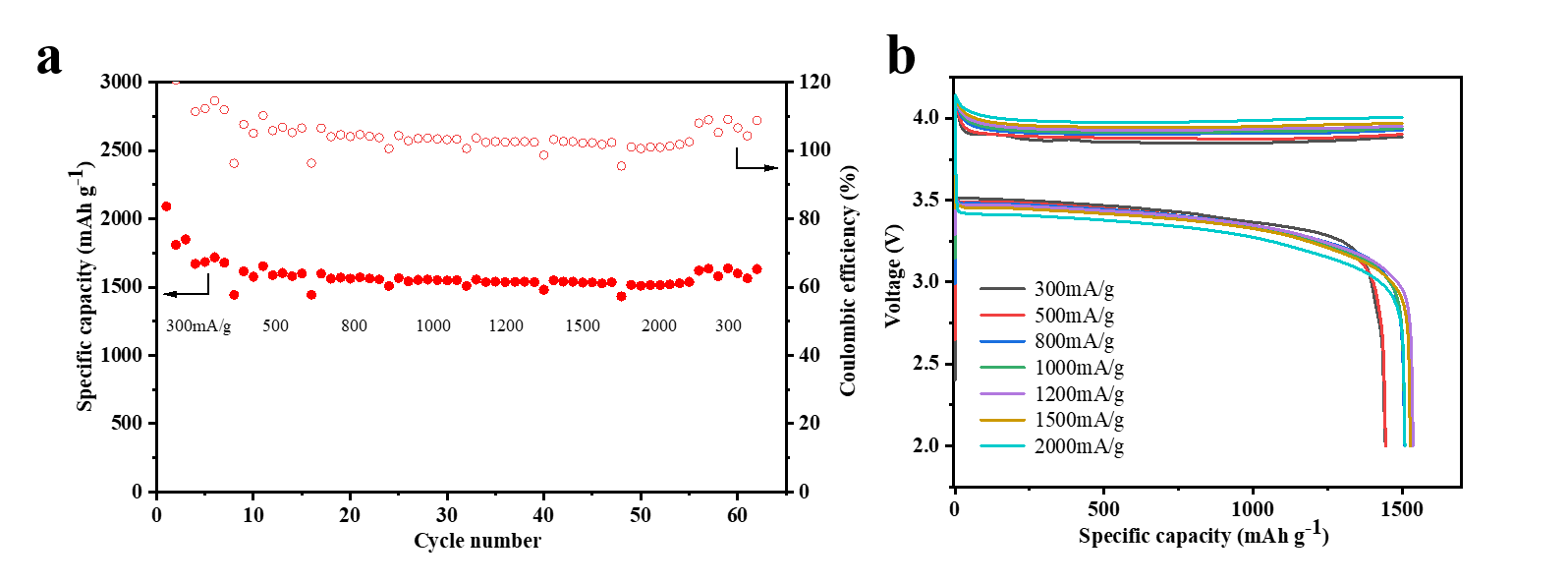


**Figure S16.** The cycling performance and voltage profiles of Li-Cl_2_@Al_2_O_3_@rGO cell under different current densities.


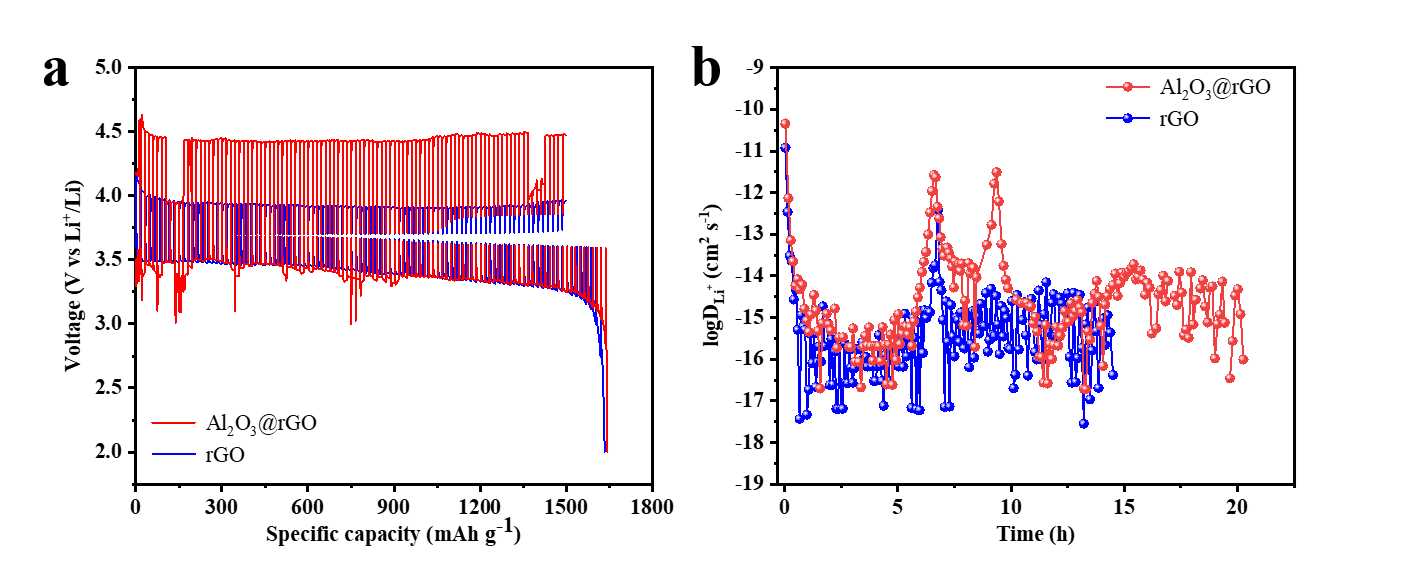


**Figure S17.** (a) The GITT curves of rGO and Al_2_O_3_@rGO and (b) the evolution of lithium-ion diffusion coefficients during charge/discharge processes.


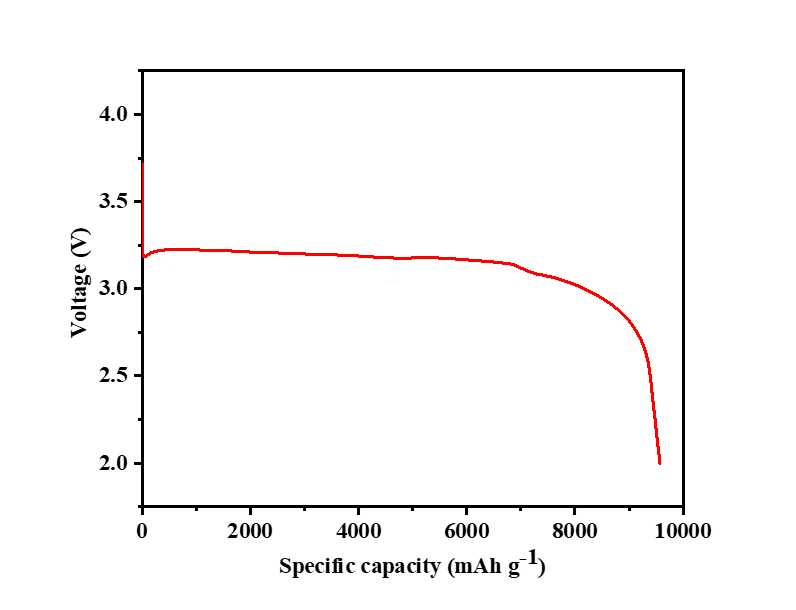


**Figure S18.** Voltage profile of Li-Cl_2_@Al_2_O_3_@rGO cell in the first cycle at -20℃.

[1] P. Hohenberg, W. Kohn, *Phys. Rev.* **1964**, *136*, B864.

[2] W. Kohn, L. J. Sham, *Phys. Rev.* **1965**, *140*, A1133.

[3] G. Kresse, J. Furthmüller, *Phys. Rev. B* **1996**, *54*, 11169.

[4] P. E. Blöchl, *Phys. Rev. B* **1994**, *50*, 17953.

[5] J. P. Perdew, K. Burke, M. Ernzerhof, *Phys. Rev. Lett.* **1996**, *77*, 3865.

[6] S. Grimme, J. Antony, S. Ehrlich, H. Krieg, *J. Chem. Phys.* **2010**, *132*, 154104.

[7] J. Xiao, D. Mei, X. Li, W. Xu, D. Wang, G. L. Graff, W. D. Bennett, Z. Nie, L. V. Saraf, I. A. Aksay, J. Liu, J.-G. Zhang, *Nano Lett.* **2011**, *11*, 5071.
